# Supplementary material for: The Tip of Brucella O-Polysaccharide Is a Potent Epitope in Response to Brucellosis Infection and Enables Short Synthetic Antigens to Be Superior Diagnostic Reagents
Source: Microorganisms. 2022 Mar 25;10(4):708. doi: 10.3390/microorganisms10040708 (PMC9024974; doi:10.3390/microorganisms10040708)
Supplement: Supplementary file 1 [file microorganisms-10-00708-s001.zip › Duncombe et al Supplementary figures S1 to S17 Reviewed 22.3.22.pdf]

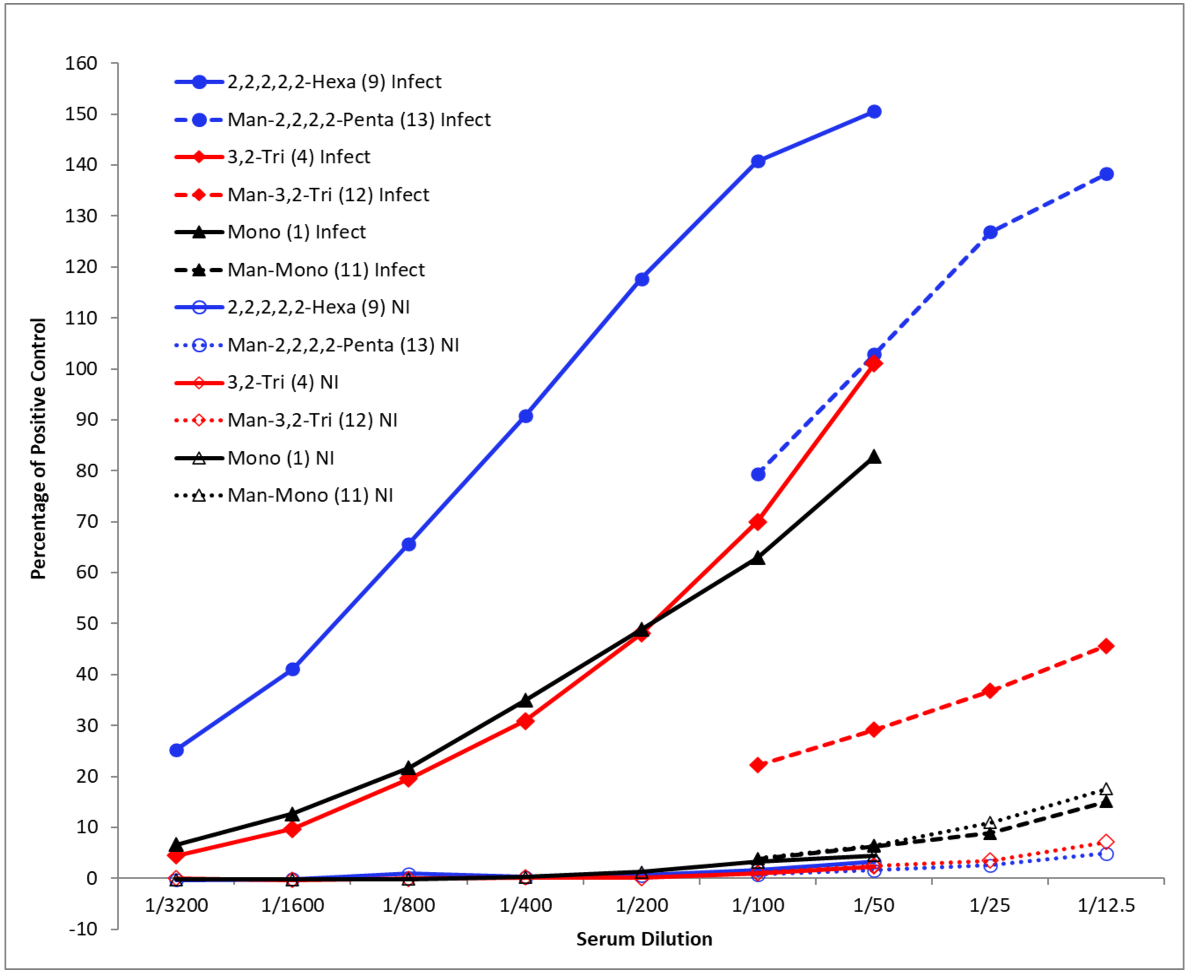

Figure S1. Line graph showing the average points on the dose response curves for 6 different positive sera (derived from GB) (Infect) and 2 different negative sera (Non-Infected, NI) when tested against six different synthetic antigens (conjugated to BSA via squarate linker). The antigens are paired into capped (caps are mannose) and non-capped equivalents and each pair is indicated by colour with results for the non-capped antigen shown as a solid line and the capped antigen as a dashed line. Results for the positive sera are shown as filled data points and results for the negative sera are shown as open data points. Serum dilution is shown on the x-axis. The response is shown as a percentage of the response to a common positive control applied to the 2,3,2-Tetra **(6)** antigen.

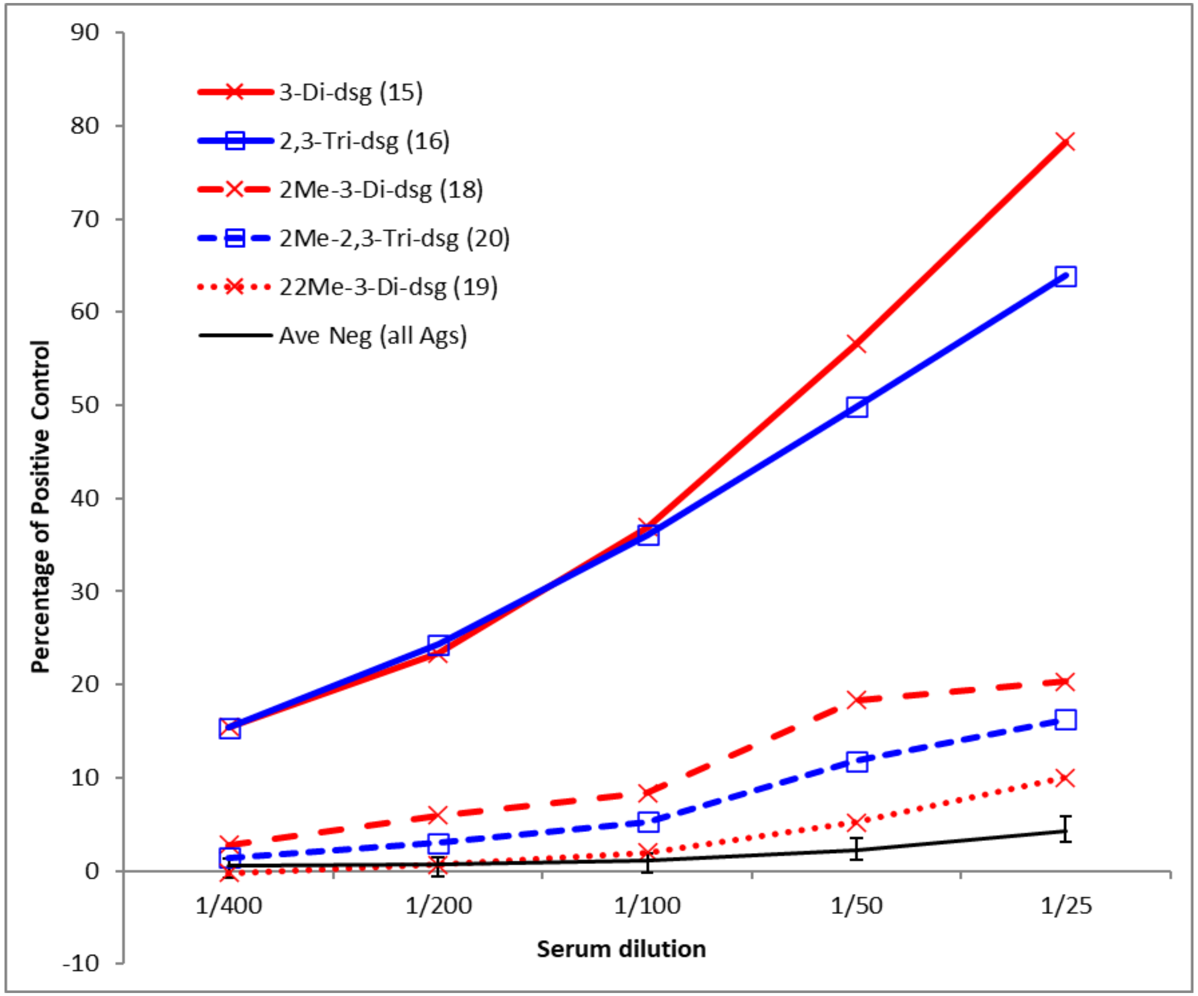

Figure S2. Line graph showing the average points on the dose response curves for 6 different positive sera (derived from GB) and 2 different negative sera when tested against five different synthetic antigens (conjugated to BSA via DSG linker). The antigens are grouped into capped (caps are methyl) and non-capped equivalents and each group is indicated by colour with results for the non-capped antigen shown as a solid line and the singly capped antigens as a dashed line and doubly capped antigen as a dotted line. Results for the

positive sera are shown as blue or red lines and the results for the negative sera against all antigens are shown as a single black line with maximum and minimum bars. Serum dilution is shown on the x-axis. The response is shown as a percentage of the response to a common positive control applied to the 2,3,2-Tetra **(6)** antigen.

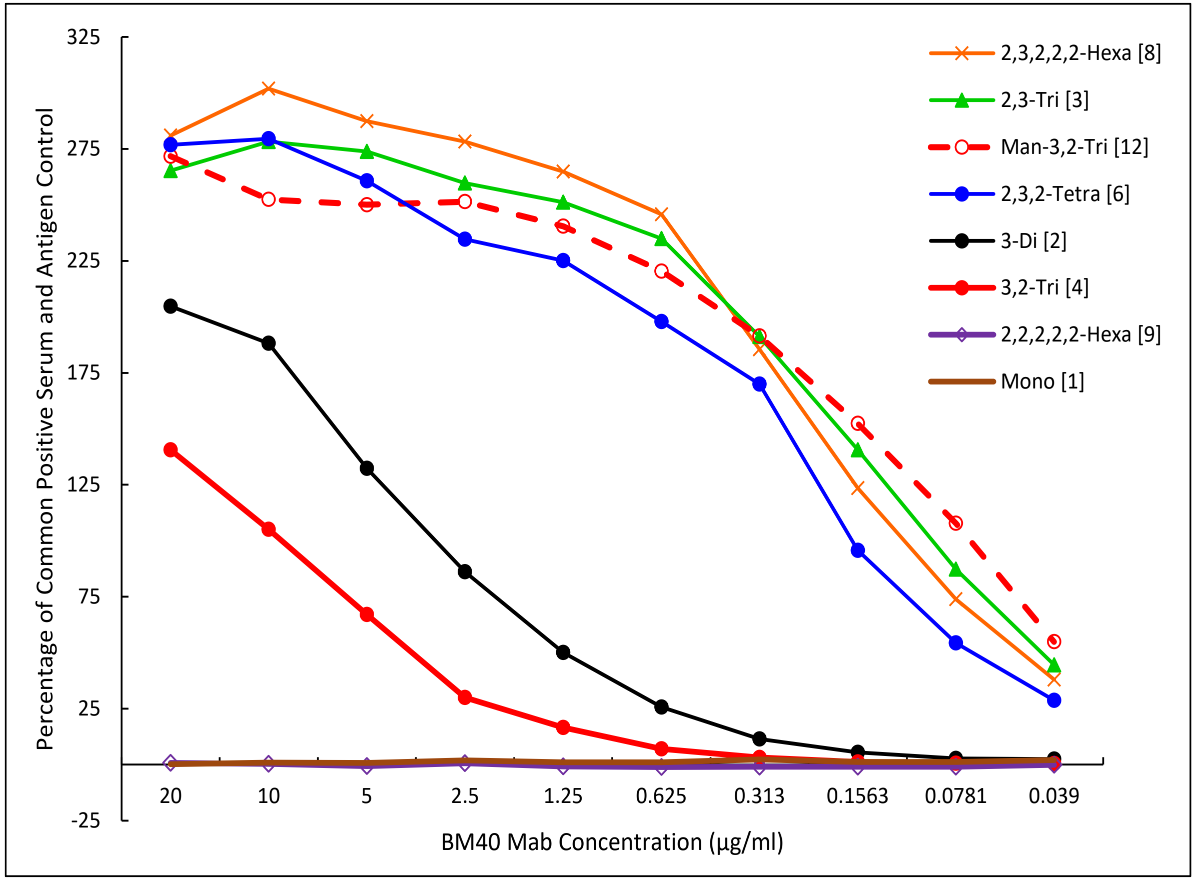

Figure S3. M epitope specific MAb (BM40) binding to synthetic oligosaccharide conjugate antigens. The results (y-axis) are expressed as a percentage of a common positive control sample (BM40 at 5 µg/ml) and antigen (*B. melitensis* 16M sLPS). The concentration of MAb is shown on the x-axis. The synthetic antigens are shown in the legend and are: 2,3,2,2,2-Hexa **(8)** (orange), 2,3-Tri **(3)** (green), Man-3,2-Tri **(12)** (dashed red line), 2,3,2-Tetra **(6)** (blue), 3-Di **(2)** (black), 3,2-Tri **(4)** (red), 2,2,2,2,2-Hexa **(9)** (purple) and Mono **(1)** (brown).

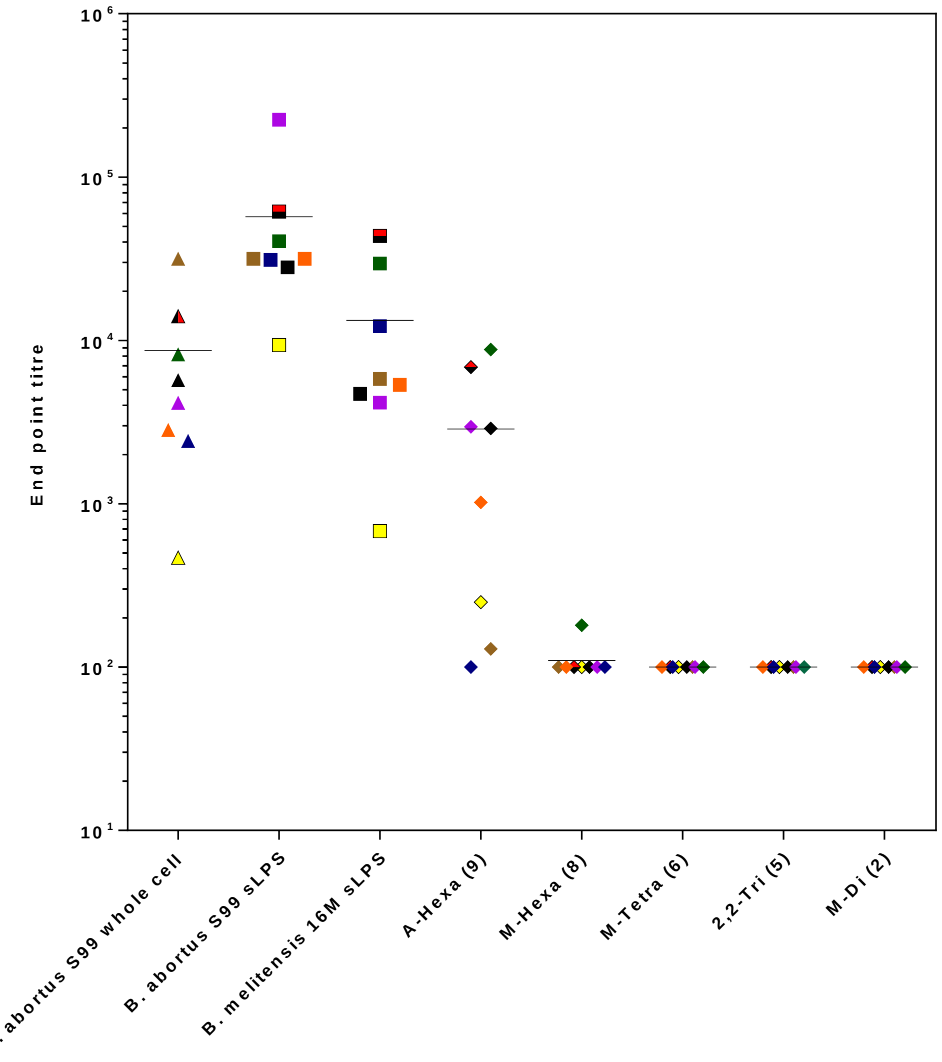

Figure S4. Results from iELISA showing end-point titres for different antigens (as described on x-axis) as applied to sera derived from mice immunised with *B. abortus* OPS tetanus conjugate. With the exception of the results for the 2,2-Tri **(5),** which were generated in this study, the results are as described previously [14].

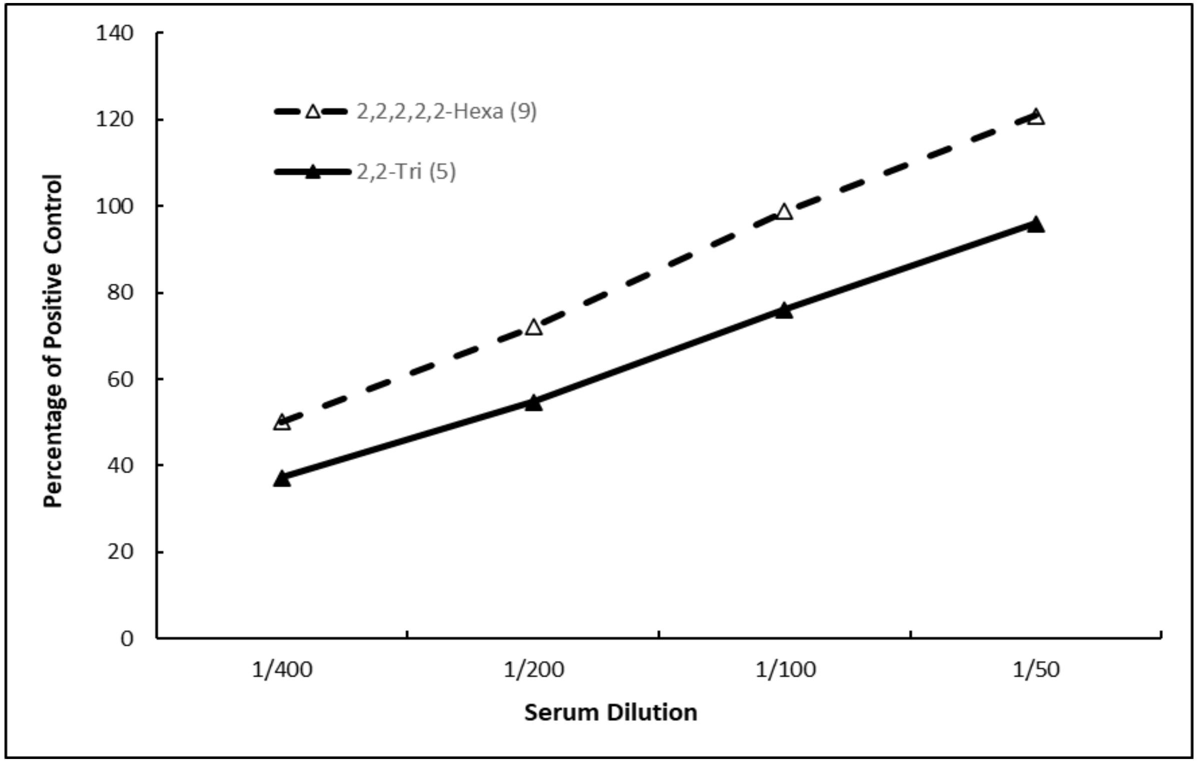

Figure S5. Average results from cattle sera collected at weeks 16 and 24 post infection with *B. abortus* (n = 8 samples) tested with 2,2,2,2,2-Hexa **(9)** (dashed line) and 2,2-Tri **(5)** (solid line). The response is shown as a percentage of the response to a common positive control applied to the 2,3,2-Tetra **(6)** antigen.

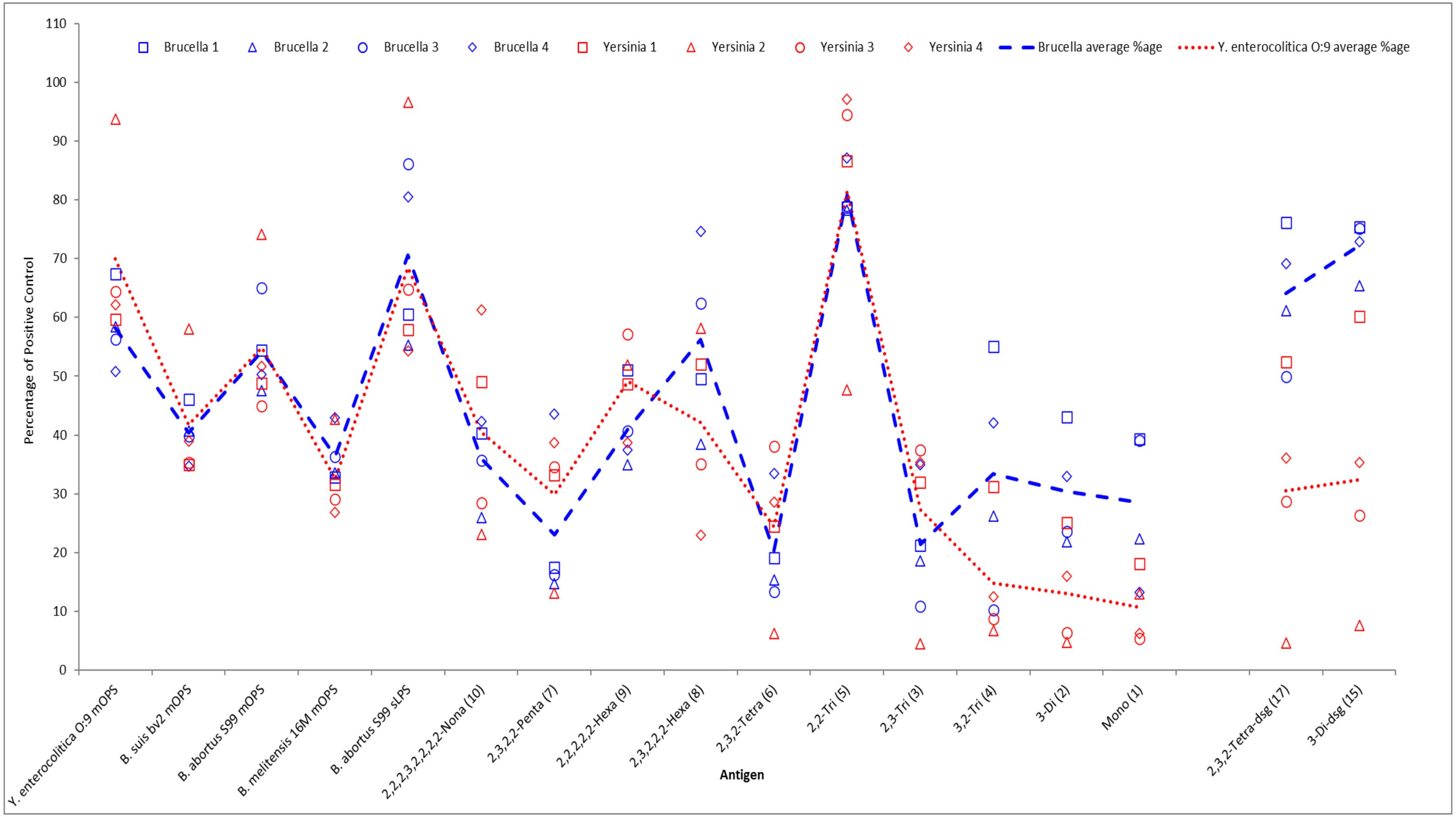

Figure S6. Sera collected at 3 weeks post-infection from four cattle experimentally infected with *B. abortus* strain 544 and four infected with *Y. enterocolitica* O:9 evaluated by iELISA. The sera were tested against 21 different antigens, 4 modified OPS antigens, one sLPS antigen and 16 synthetic antigens, as described on the x-axis. Individual data points are shown

for sera from each animal, *B. abortus* infected in blue and *Y. enterocolitica* O:9 infected in red. The average results from the 4 samples from each infection type are shown by the dotted lines (*B. abortus* in blue, *Y. enterocolitica* O:9 in red). The iELISA results are expressed as a percentage of a common positive control that was applied to each antigen type.

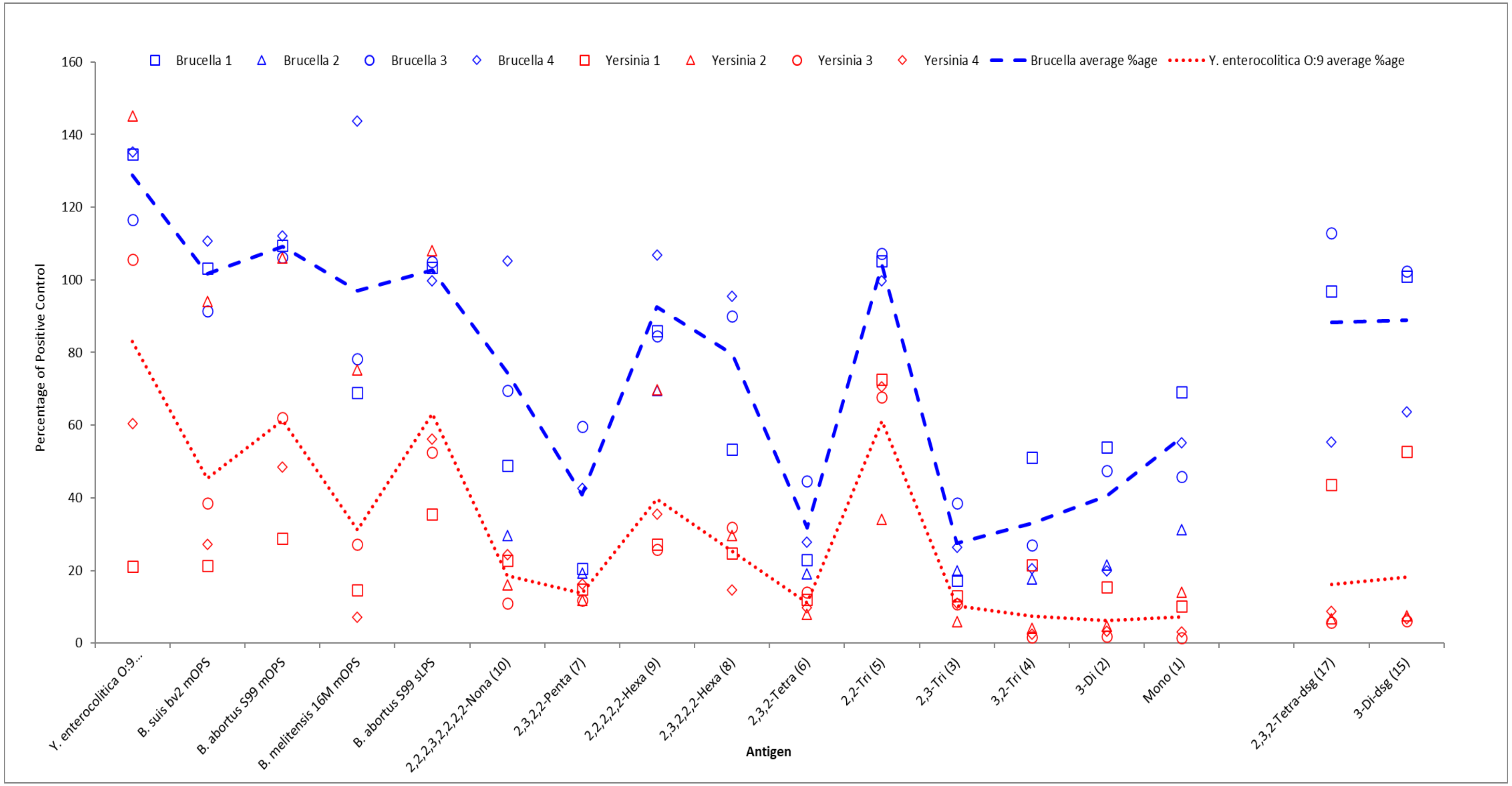

Figure S7. Sera collected at 7 weeks post-infection from four cattle experimentally infected with *B. abortus* strain 544 and four infected with *Y. enterocolitica* O:9 evaluated by iELISA. The sera were tested against 21 different antigens, 4 modified OPS antigens, one sLPS antigen and 16 synthetic antigens, as described on the x-axis. Individual data points are shown for sera from each animal, *B. abortus* infected in blue and *Y. enterocolitica* O:9 infected in red. The average results from the 4 samples from each infection type are shown by the dotted lines (*B. abortus* in blue, *Y. enterocolitica* O:9 in red). The iELISA results are expressed as a percentage of a common positive control that was applied to each antigen type.

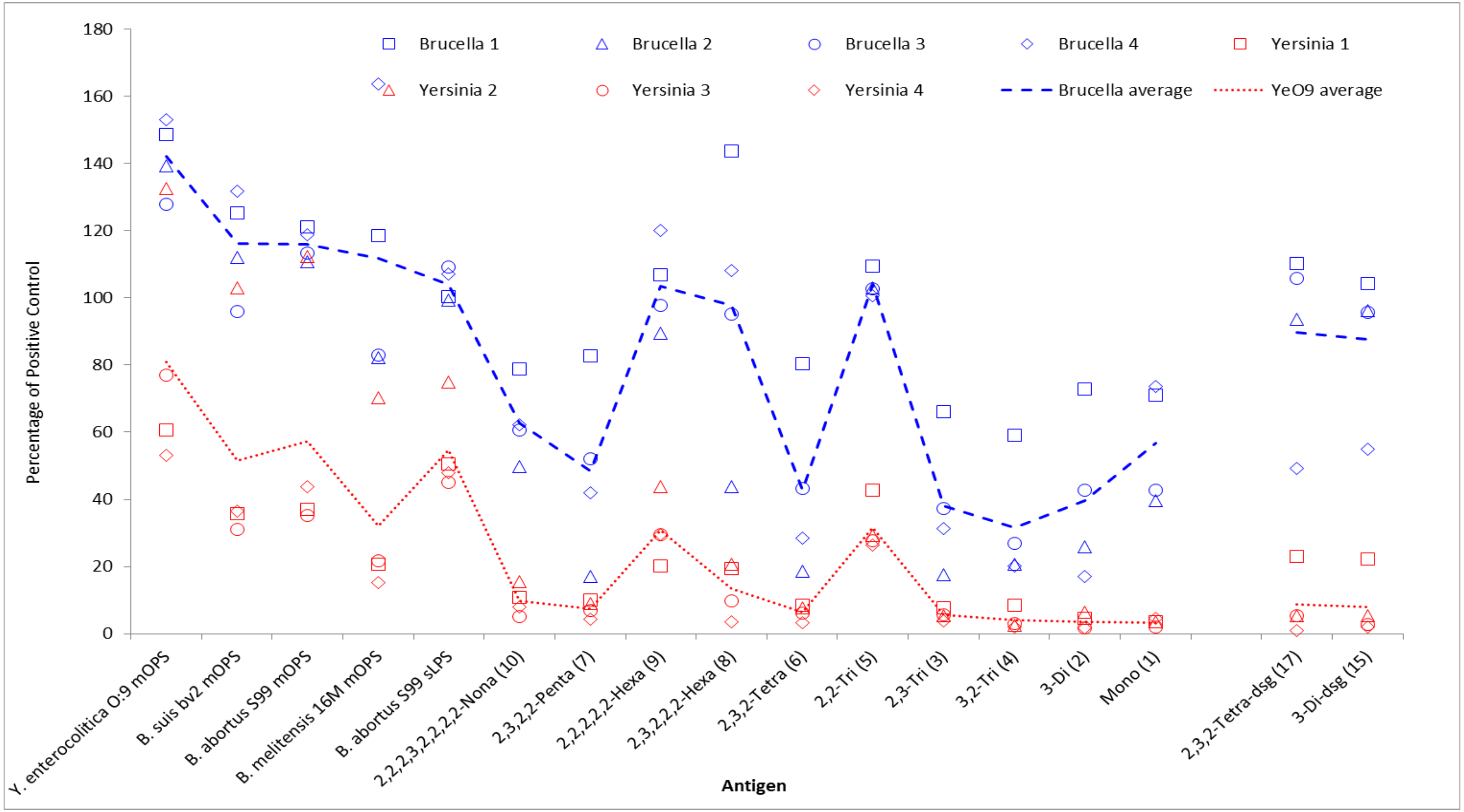

Figure S8 Sera collected at 16 weeks post-infection from four cattle experimentally infected with *B. abortus* strain 544 and four infected with *Y. enterocolitica* O:9 evaluated by iELISA. The sera were tested against 21 different antigens, 4 modified OPS antigens, one sLPS antigen and 16 synthetic antigens, as described on the x-axis. Individual data points are shown

for sera from each animal, *B. abortus* infected in blue and *Y. enterocolitica* O:9 infected in red. The average results from the 4 samples from each infection type are shown by the dotted lines (*B. abortus* in blue, *Y. enterocolitica* O:9 in red). The iELISA results are expressed as a percentage of a common positive control that was applied to each antigen type.

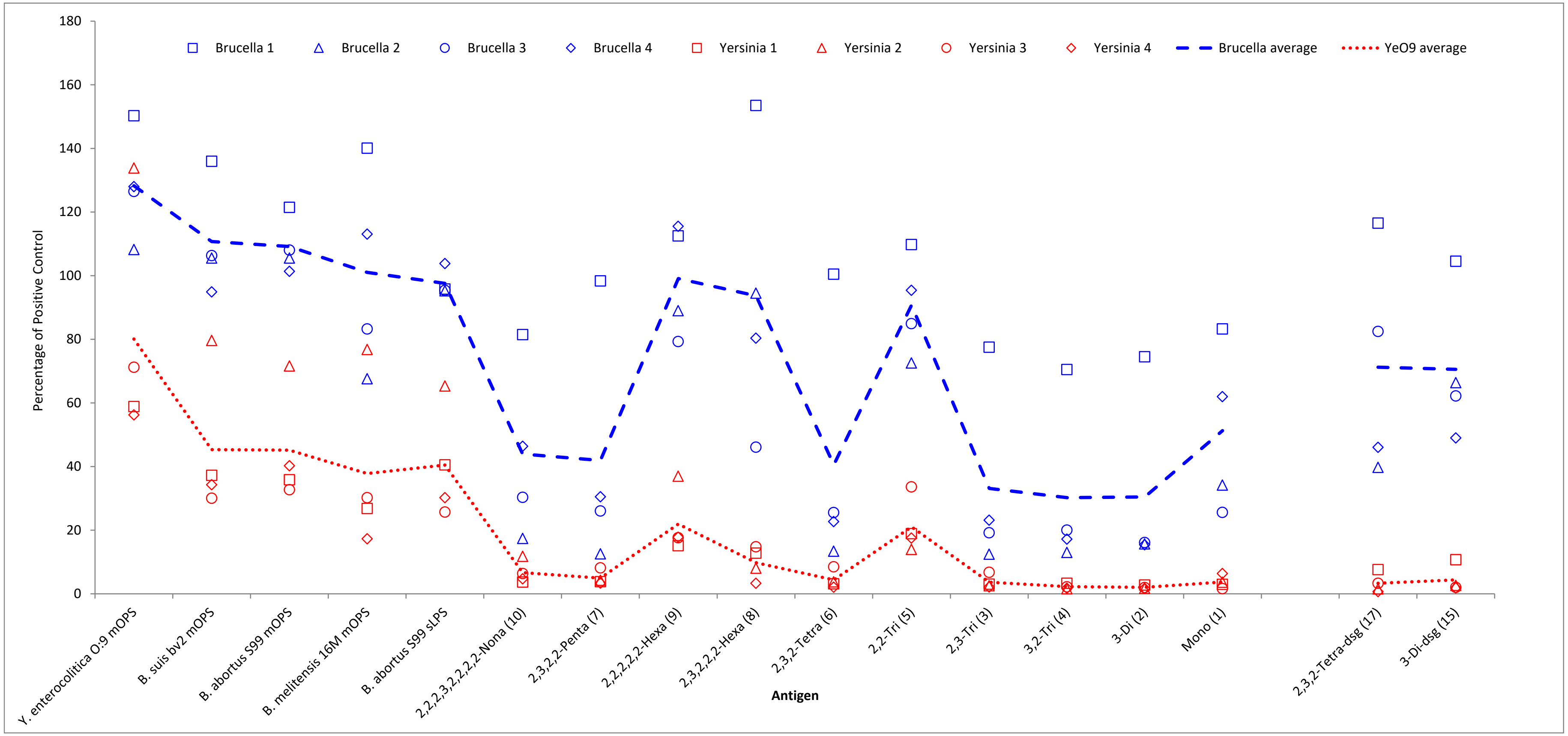

Figure S9. Sera collected at 24 weeks post-infection from four cattle experimentally infected with *B. abortus* strain 544 and four infected with *Y. enterocolitica* O:9 evaluated by iELISA. The sera were tested against 21 different antigens, 4 modified OPS antigens, one sLPS antigen and 16 synthetic antigens, as described on the x-axis. Individual data points are shown for sera from each animal, *B. abortus* infected in blue and *Y. enterocolitica* O:9 infected in red. The average results from the 4 samples from each infection type are shown by the dotted lines (*B. abortus* in blue, *Y. enterocolitica* O:9 in red). The iELISA results are expressed as a percentage of a common positive control that was applied to each antigen type.

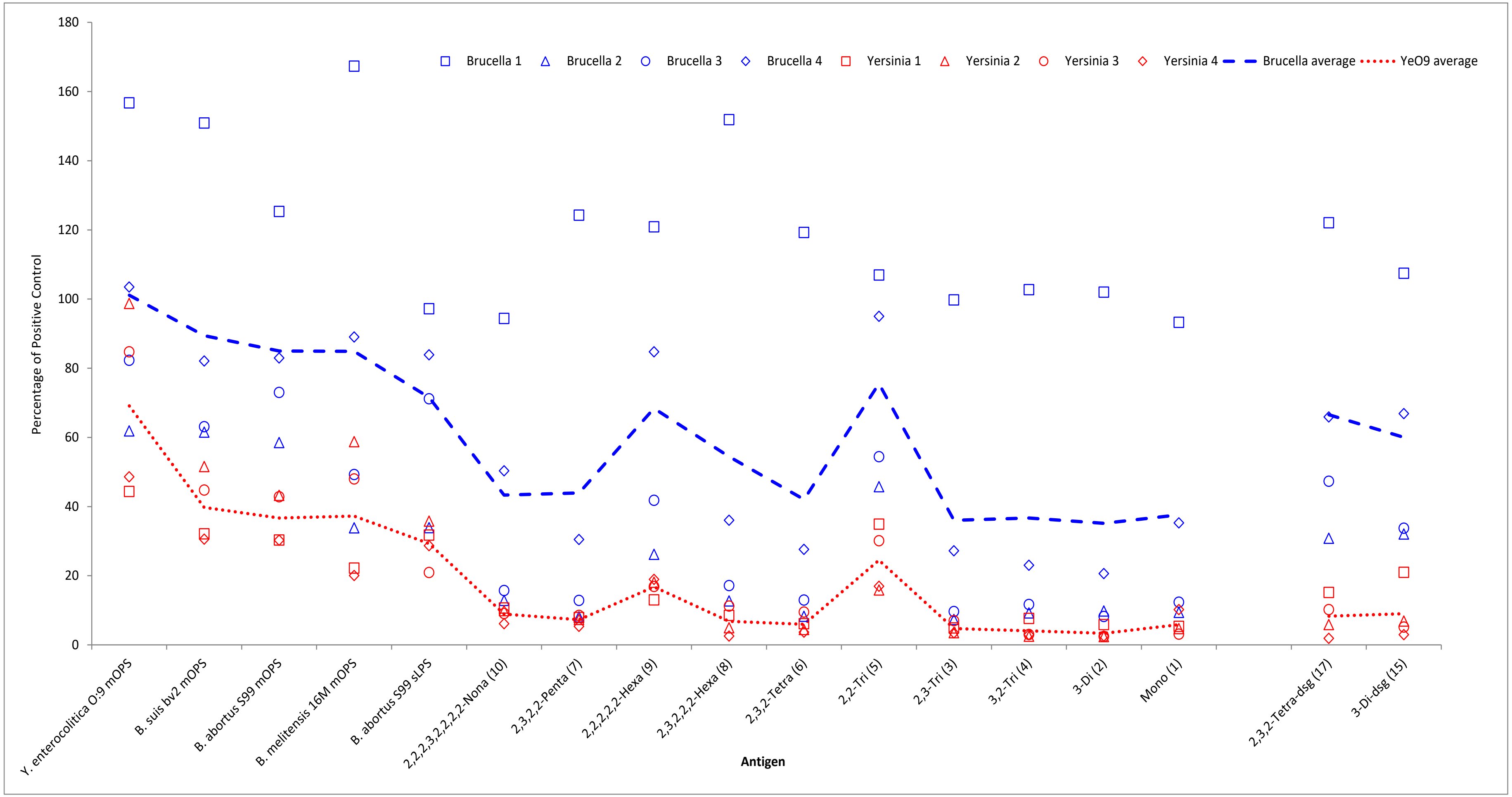

Figure S10. Sera collected at 53 weeks post-infection from four cattle experimentally infected with *B. abortus* strain 544 and four infected with *Y. enterocolitica* O:9 evaluated by iELISA. The sera were tested against 21 different antigens, 4 modified OPS antigens, one sLPS antigen and 16 synthetic antigens, as described on the x-axis. Individual data points are shown for sera from each animal, *B. abortus* infected in blue and *Y. enterocolitica* O:9 infected in red. The average results from the 4 samples from each infection type are shown by the dotted lines (*B. abortus* in blue, *Y. enterocolitica* O:9 in red). The iELISA results are expressed as a percentage of a common positive control that was applied to each antigen type.

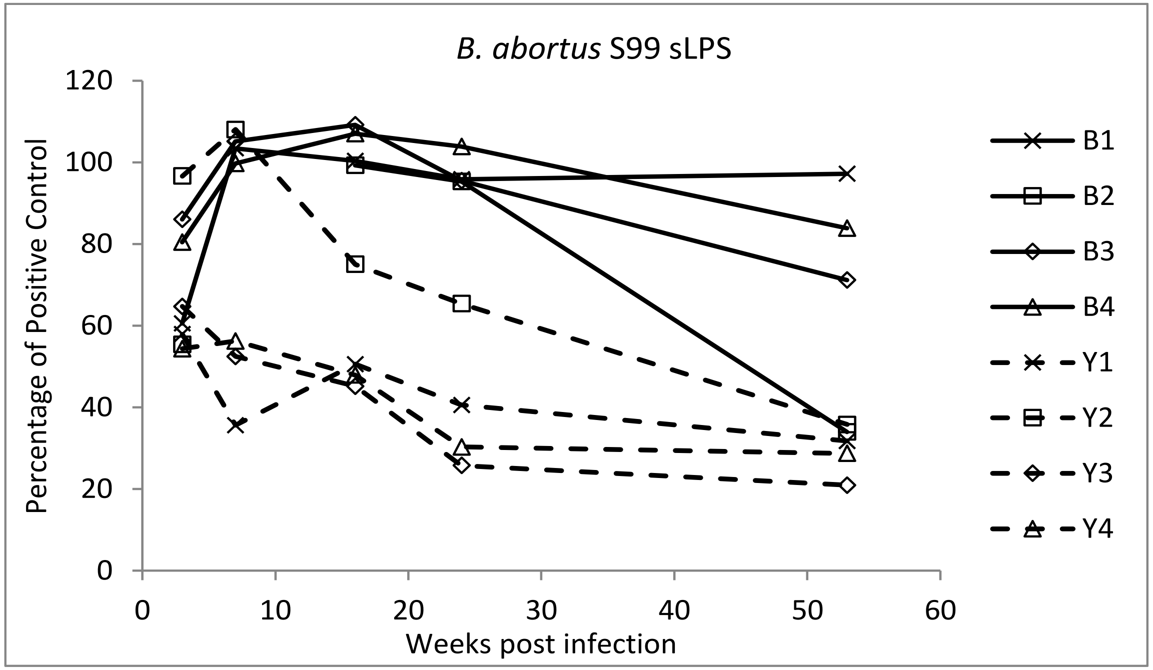

Figure S11. Results from iELISA performed with sera collected from four cattle experimentally infected with *B. abortus* strain 544 and four infected with *Y. enterocolitica* O:9 at five sampling dates (3, 7, 16, 24 and 53 weeks post infection) as show in the x-axis. The sera were tested against *B. abortus* strain S99 sLPS. Individual data points are shown for sera from each animal, *B. abortus* as nodes linked by solid lines (B1 - B4 representing the 4 *Brucella* infected cattle) *Y. enterocolitica* O:9 infected as node linked by dashed lines (Y1 - Y4 representing the 4 *Brucella* infected cattle). The iELISA results (y-axis) are expressed as a percentage of a common positive control that was applied to each antigen type.

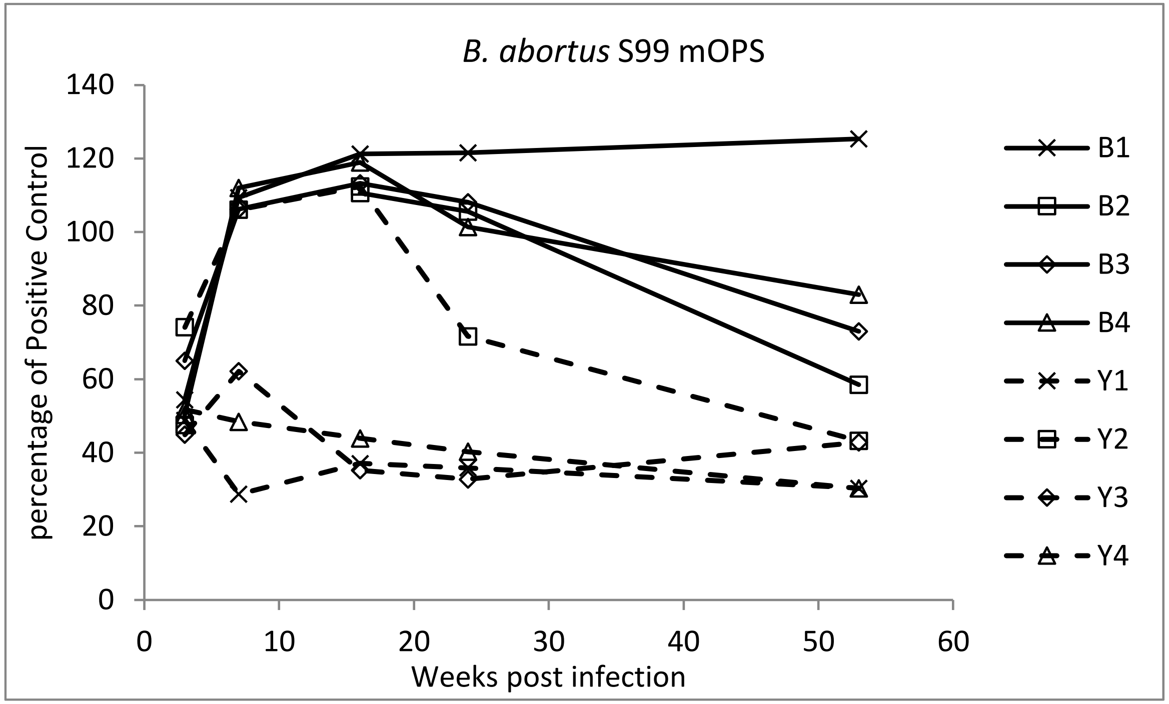

Figure S12. Results from iELISA performed with sera collected from four cattle experimentally infected with *B. abortus* strain 544 and four infected with *Y. enterocolitica* O:9 at five sampling dates (3, 7, 16, 24 and 53 weeks post infection) as show in the x-axis. The sera were tested against *B. abortus* strain S99 modified (m) OPS. Individual data points are shown for sera from each animal, *B. abortus* as nodes linked by solid lines (B1 - B4 representing the 4 *Brucella* infected cattle) *Y. enterocolitica* O:9 infected as node linked by dashed lines (Y1 - Y4 representing the 4 *Brucella* infected cattle). The iELISA results (y-axis) are expressed as a percentage of a common positive control that was applied to each antigen type.

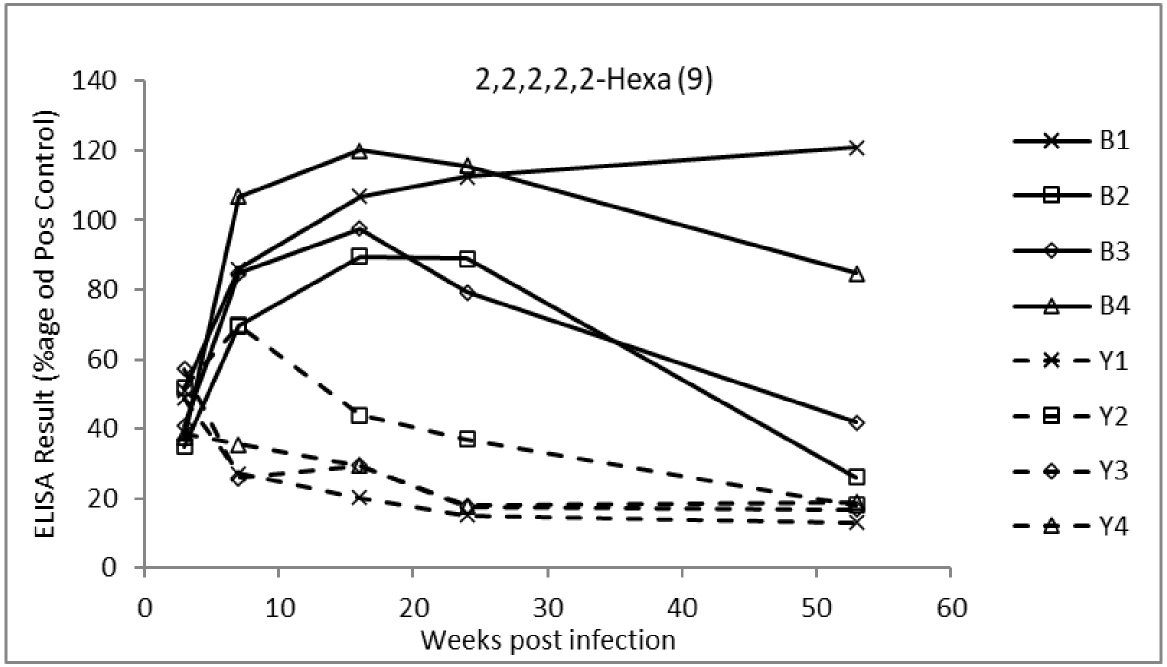

Figure S13. Results from iELISA performed with sera collected from four cattle experimentally infected with *B. abortus* strain 544 and four infected with *Y. enterocolitica* O:9 at five sampling dates (3, 7, 16, 24 and 53 weeks post infection) as show in the x-axis. The sera were tested against the 2,2,2,2,2-Hexa **(9)** antigen. Individual data points are shown for sera from each animal, *B. abortus* as nodes linked by solid lines (B1 - B4 representing the 4 *Brucella* infected cattle) *Y. enterocolitica* O:9 infected as node linked by dashed lines (Y1 - Y4 representing the 4 *Brucella* infected cattle). The iELISA results (y-axis) are expressed as a percentage of a common positive control that was applied to each antigen type.

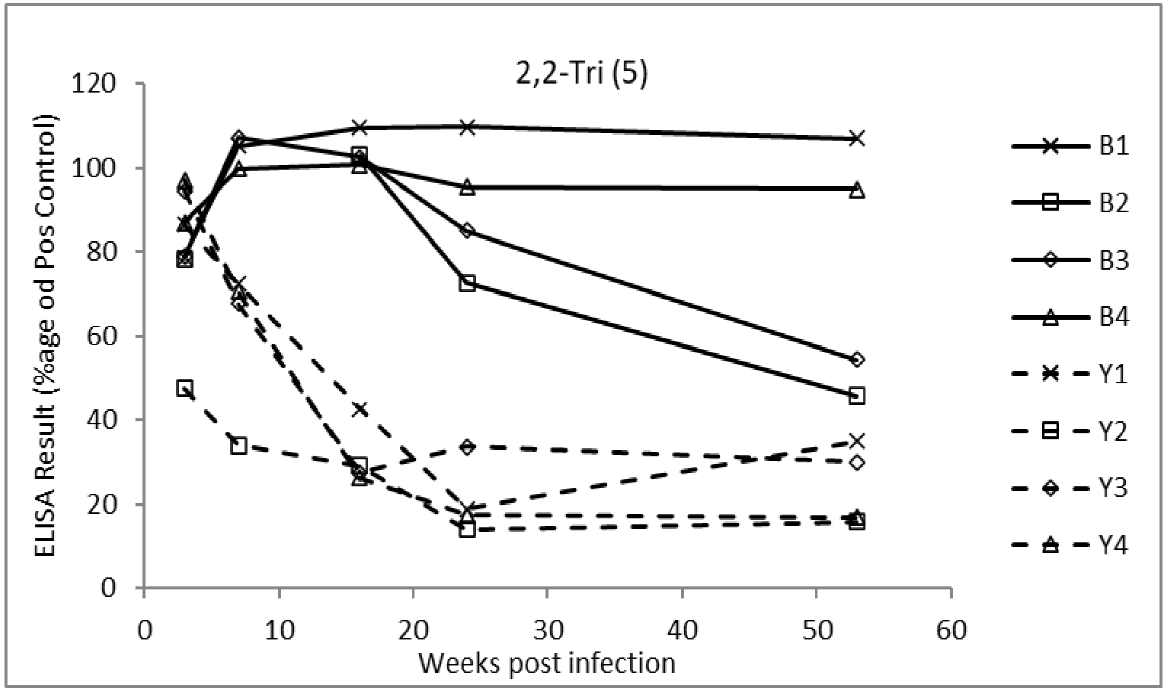

Figure S14. Results from iELISA performed with sera collected from four cattle experimentally infected with *B. abortus* strain 544 and four infected with *Y. enterocolitica* O:9 at five sampling dates (3, 7, 16, 24 and 53 weeks post infection) as show in the x-axis. The sera were tested against the 2,2-Tri **(5)** antigen. Individual data points are shown for sera from each animal, *B. abortus* as nodes linked by solid lines (B1 - B4 representing the 4 *Brucella* infected cattle) *Y. enterocolitica* O:9 infected as node linked by dashed lines (Y1 - Y4 representing the 4 *Brucella* infected cattle). The iELISA results (y-axis) are expressed as a percentage of a common positive control that was applied to each antigen type.

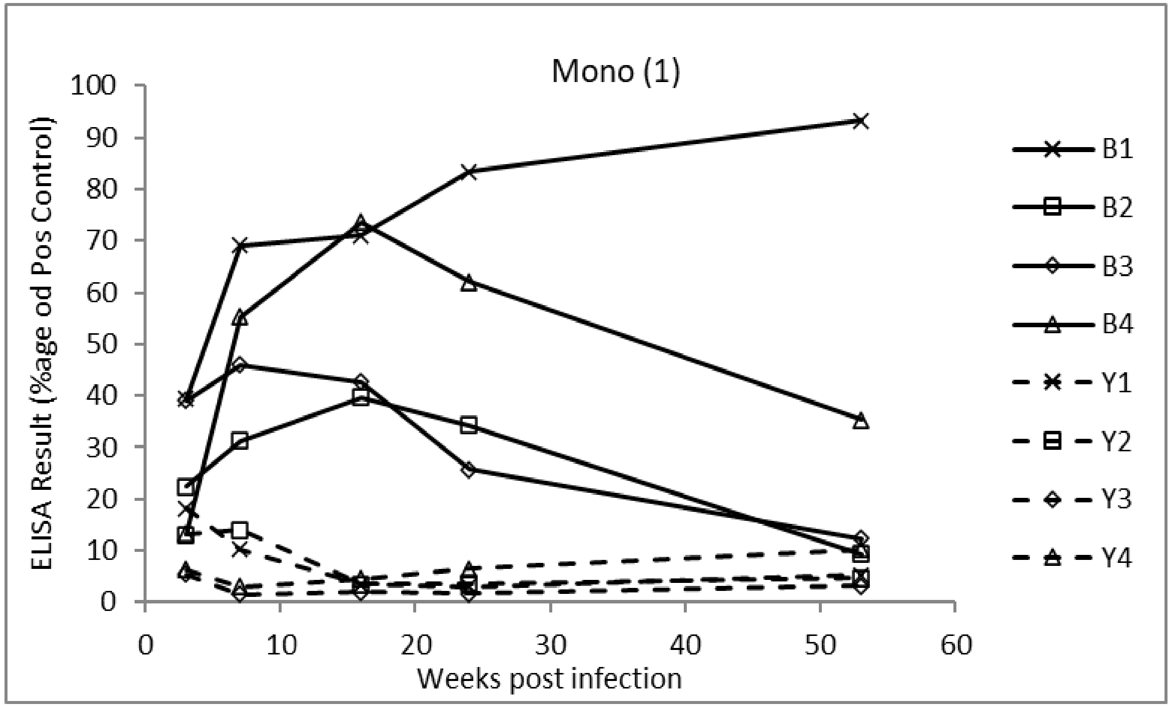

Figure S15. Results from iELISA performed with sera collected from four cattle experimentally infected with *B. abortus* strain 544 and four infected with *Y. enterocolitica* O:9 at five sampling dates (3, 7, 16, 24 and 53 weeks post infection) as show in the x-axis. The sera were tested against the Mono **(1)** antigen. Individual data points are shown for sera from each animal, *B. abortus* as nodes linked by solid lines (B1 - B4 representing the 4 *Brucella* infected cattle) *Y. enterocolitica* O:9 infected as node linked by dashed lines (Y1 - Y4 representing the 4 *Brucella* infected cattle). The iELISA results (y-axis) are expressed as a percentage of a common positive control that was applied to each antigen type.

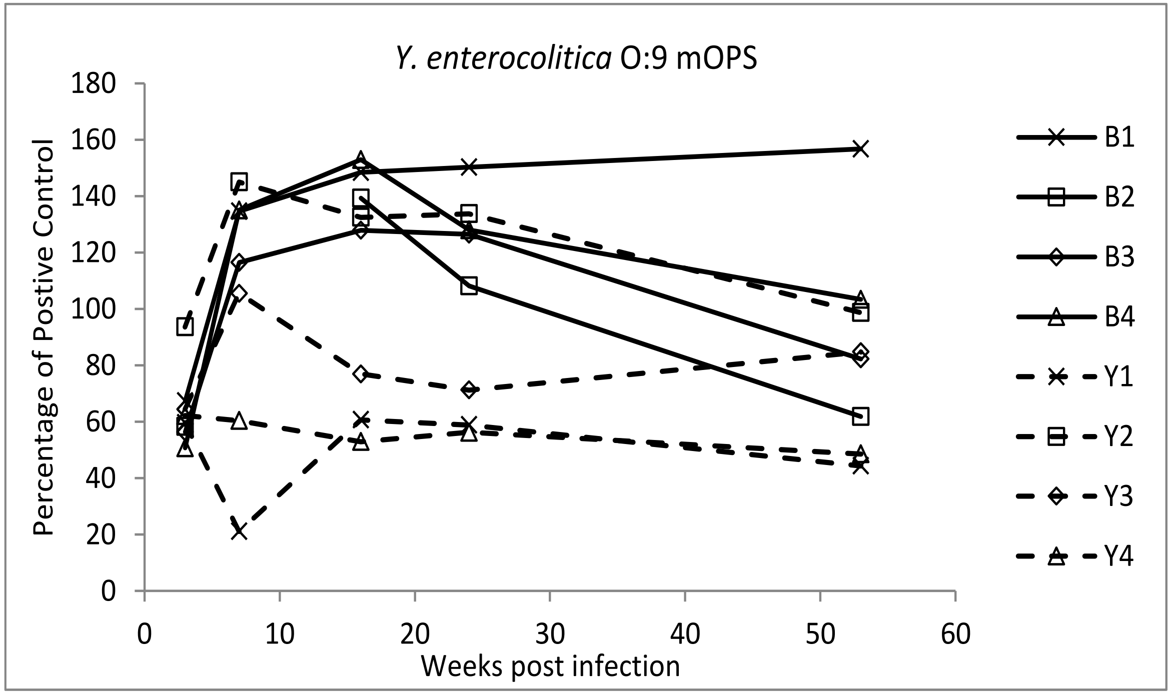

Figure S16. Results from iELISA performed with sera collected from four cattle experimentally infected with *B. abortus* strain 544 and four infected with *Y. enterocolitica* O:9 at five sampling dates (3, 7, 16, 24 and 53 weeks post infection) as show in the x-axis. The sera were tested against the *Y. enterocolitica* O:9 modified (m) OPS. Individual data points are shown for sera from each animal, *B. abortus* as nodes linked by solid lines (B1 - B4 representing the 4 *Brucella* infected cattle) *Y. enterocolitica* O:9 infected as node linked by dashed lines (Y1 - Y4 representing the 4 *Brucella* infected cattle). The iELISA results (y-axis) are expressed as a percentage of a common positive control that was applied to each antigen type.

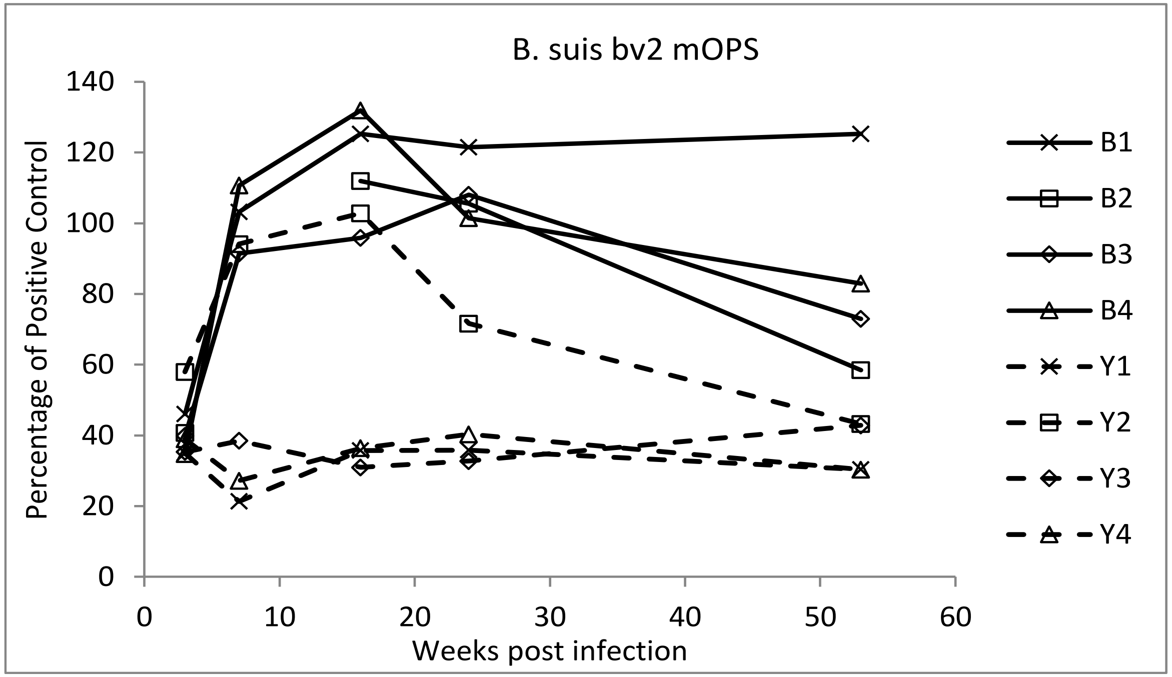

Figure S17, Results from iELISA performed with sera collected from four cattle experimentally infected with *B. abortus* strain 544 and four infected with *Y. enterocolitica* O:9 at five sampling dates (3, 7, 16, 24 and 53 weeks post infection) as show in the x-axis. The sera were tested against the *B. suis* bv2 modified (m) OPS. Individual data points are shown for sera from each animal, *B. abortus* as nodes linked by solid lines (B1 - B4 representing the 4 *Brucella* infected cattle) *Y. enterocolitica* O:9 infected as node linked by dashed lines (Y1 - Y4 representing the 4 *Brucella* infected cattle). The iELISA results (y-axis) are expressed as a percentage of a common positive control that was applied to each antigen type.
