# Supplementary material for: The Tip of Brucella O-Polysaccharide Is a Potent Epitope in Response to Brucellosis Infection and Enables Short Synthetic Antigens to Be Superior Diagnostic Reagents
Source: Microorganisms. 2022 Mar 25;10(4):708. doi: 10.3390/microorganisms10040708 (PMC9024974; doi:10.3390/microorganisms10040708)
Supplement: Supplementary file 1 [file microorganisms-10-00708-s001.zip › Duncombe et al Supplementary Scheme S1 synthetic antigen structures Reviewed.pdf]

Supplementary Scheme S1. Synthetic antigen structures 1 to 21

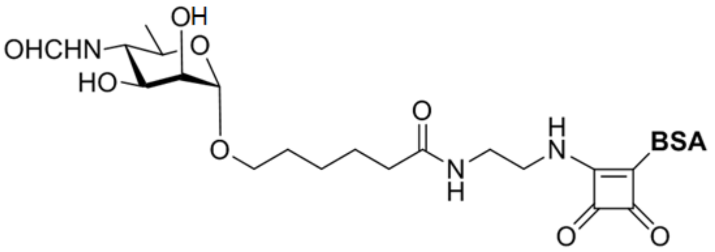

Structure **1**: Mono (α-D-Rha4NFo)

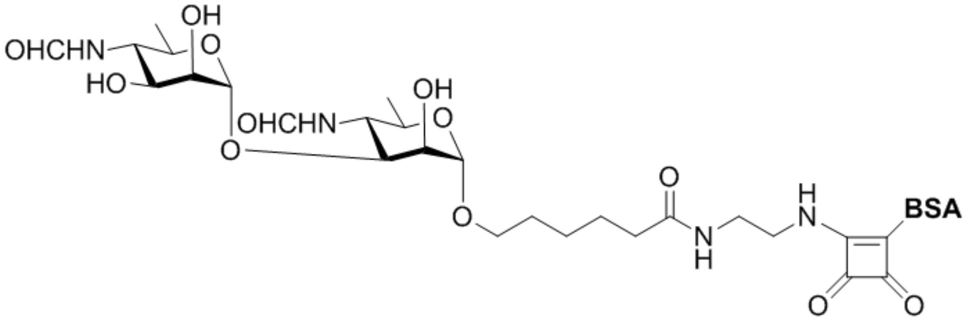

Structure **2**: 3-Di (α-D-Rha4NFo-(1→3)-α-D-Rha4NFo)

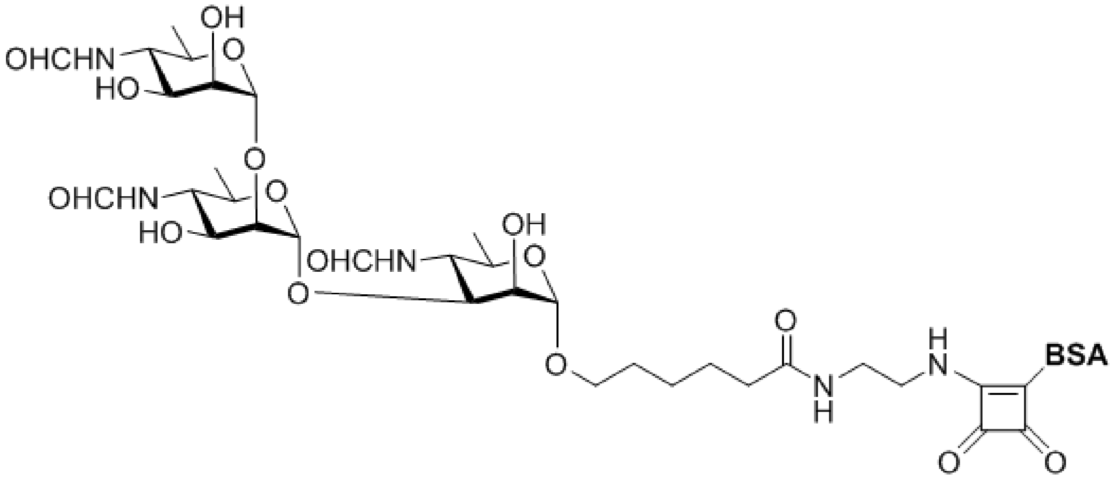

Structure **3**: 2,3-Tri (α-D-Rha4NFo-(1→2)-α-D-Rha4NFo-(1→3)-α-D-Rha4NFo)

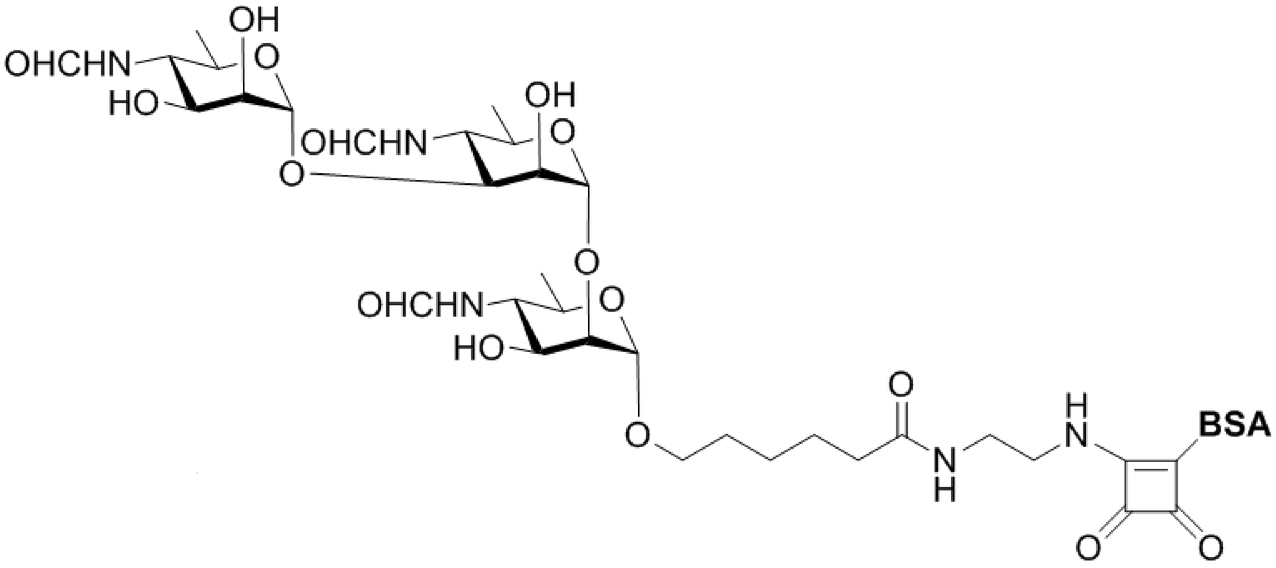

Structure **4**: 3,2-Tri (α-D-Rha4NFo-(1→3)-α-D-Rha4NFo-(1→2)-α-D-Rha4NFo)

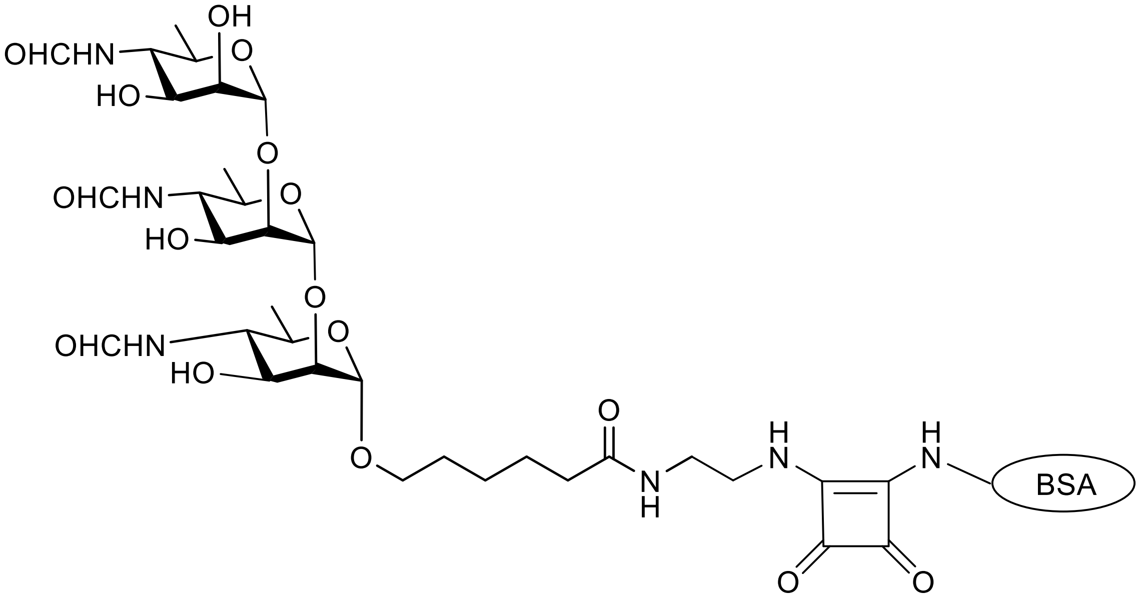

Structure **5**: 2,2-Tri (α-D-Rha4NFo-(1→2)-α-D-Rha4NFo-(1→2)-α-D-Rha4NFo)

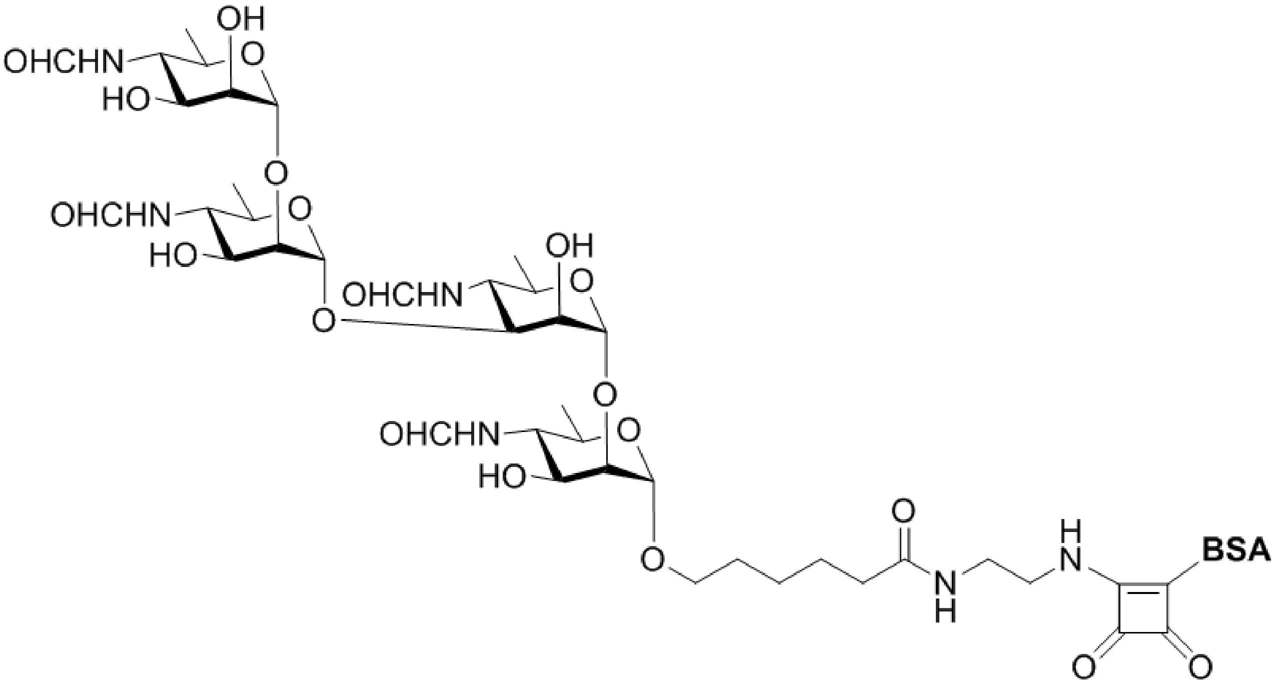

Structure **6**: 2,3,2-Tetra (α-D-Rha4NFo-(1→2)-α-D-Rha4NFo-(1→3)-α-D-Rha4NFo-(1→2)-α-D-Rha4NFo)

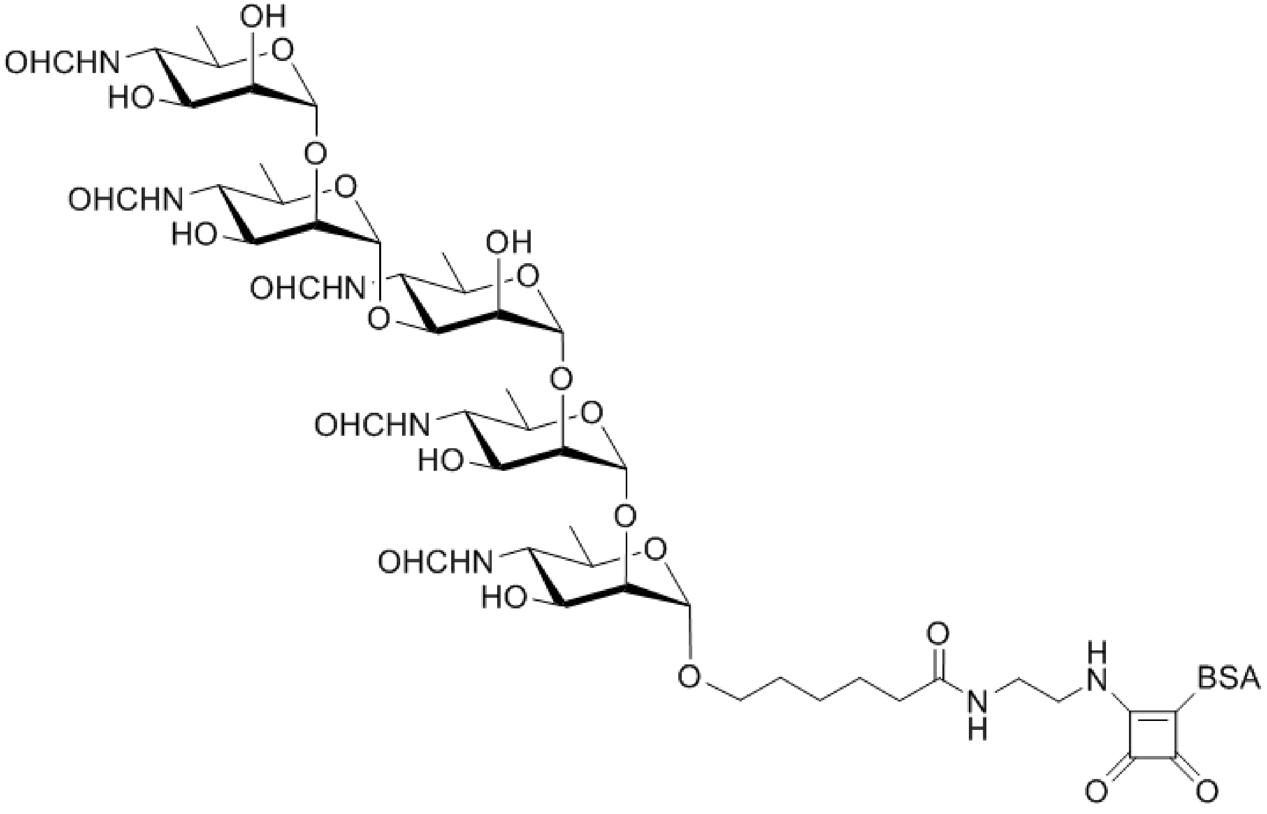

Structure **7**: 2,3,2,2-Penta (α-D-Rha4NFo-(1→2)-α-D-Rha4NFo-(1→3)-α-D-Rha4NFo-(1→2)-α-D-Rha4NFo-(1→2)- α-D-Rha4NFo)

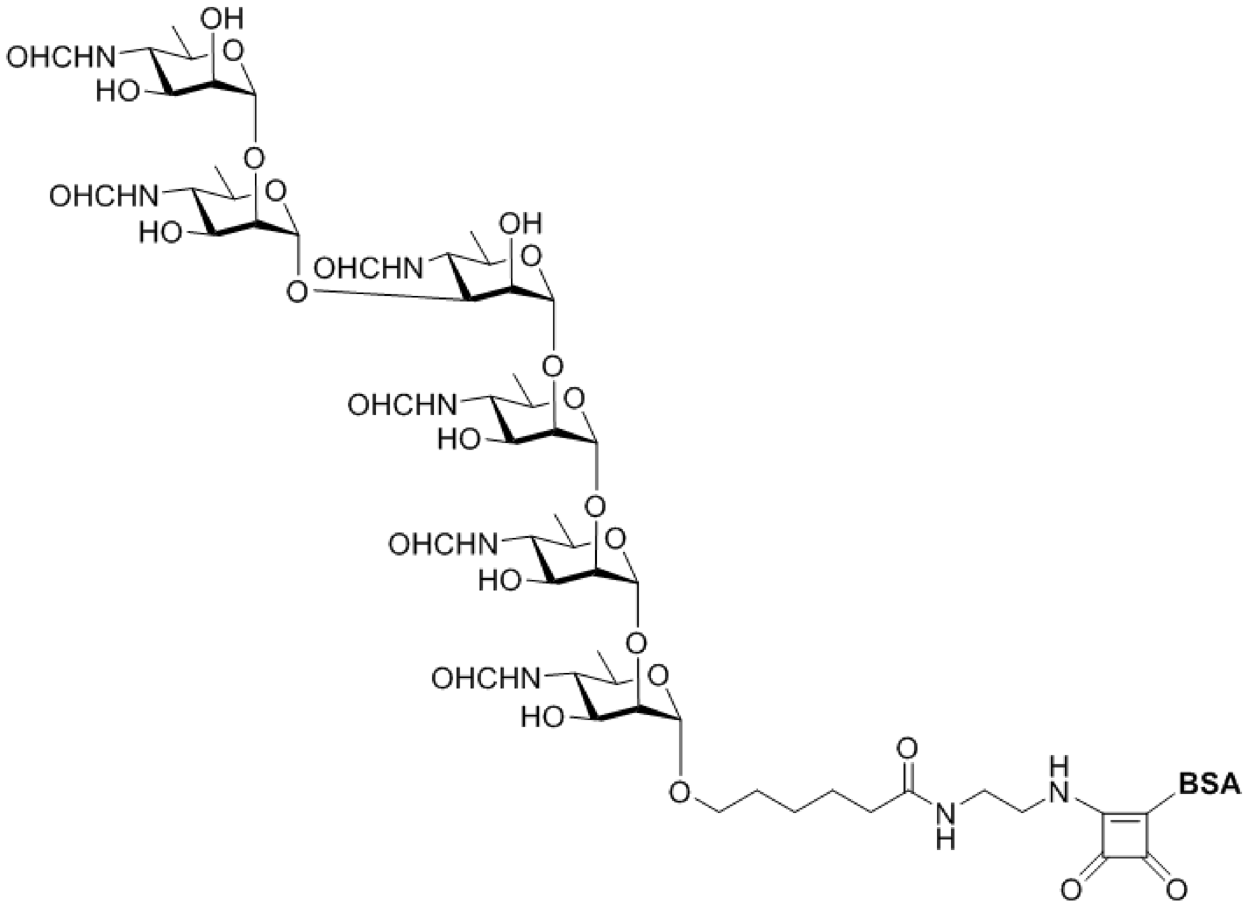

Structure **8**: 2,3,2,2,2-Hexa (α-D-Rha4NFo-(1→2)-α-D-Rha4NFo-(1→3)-α-D-Rha4NFo-(1→2)-α-D-Rha4NFo-(1→2)- α-D-Rha4NFo-(1→2)-α-D-Rha4NFo)

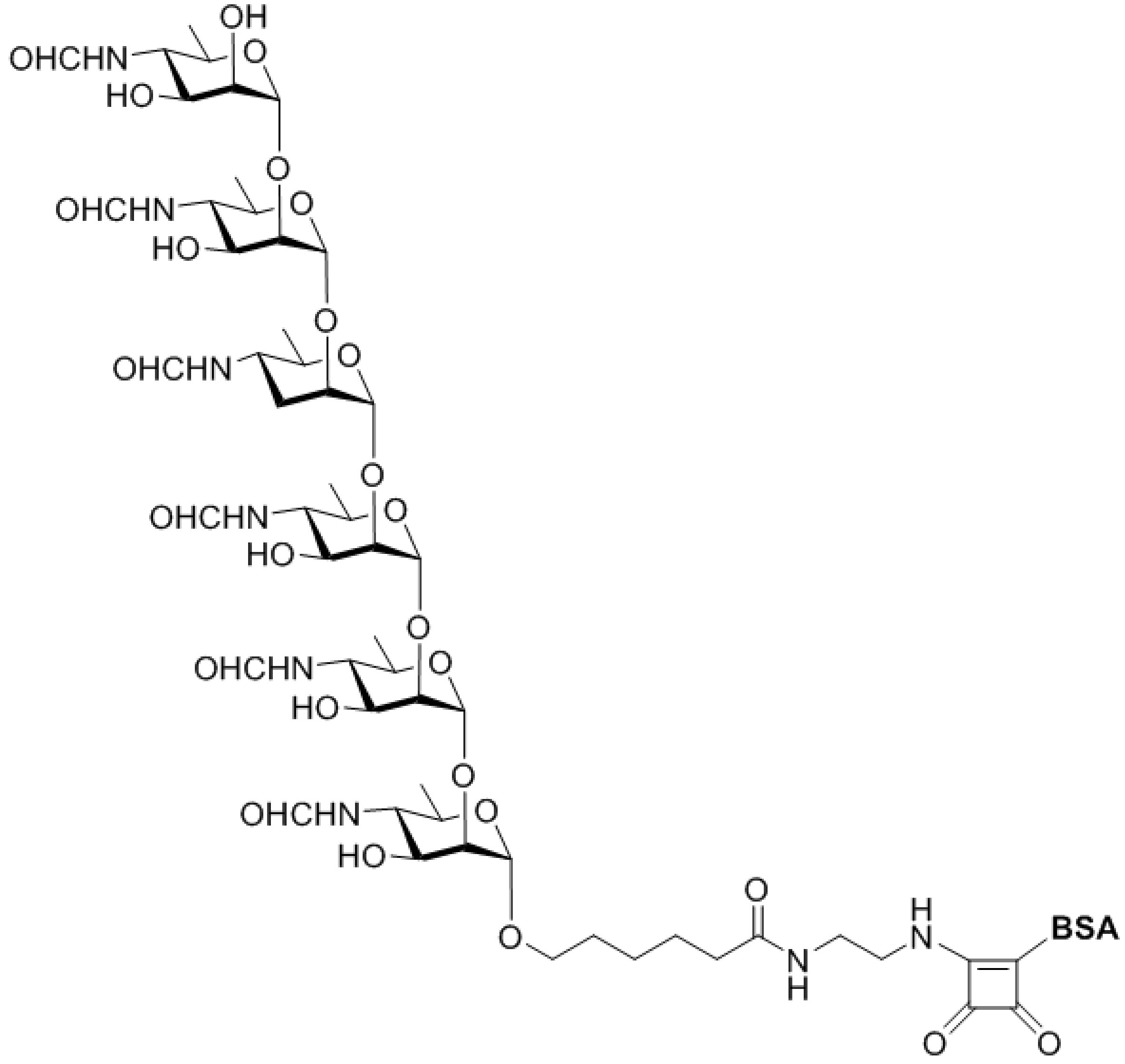

Structure **9**: 2,2,2,2,2-Hexa (α-D-Rha4NFo-(1→2)-α-D-Rha4NFo-(1→2)-α-D-Rha4NFo-(1→2)-α-D-Rha4NFo-(1→2)- α-D-Rha4NFo-(1→2)-α-D-Rha4NFo)

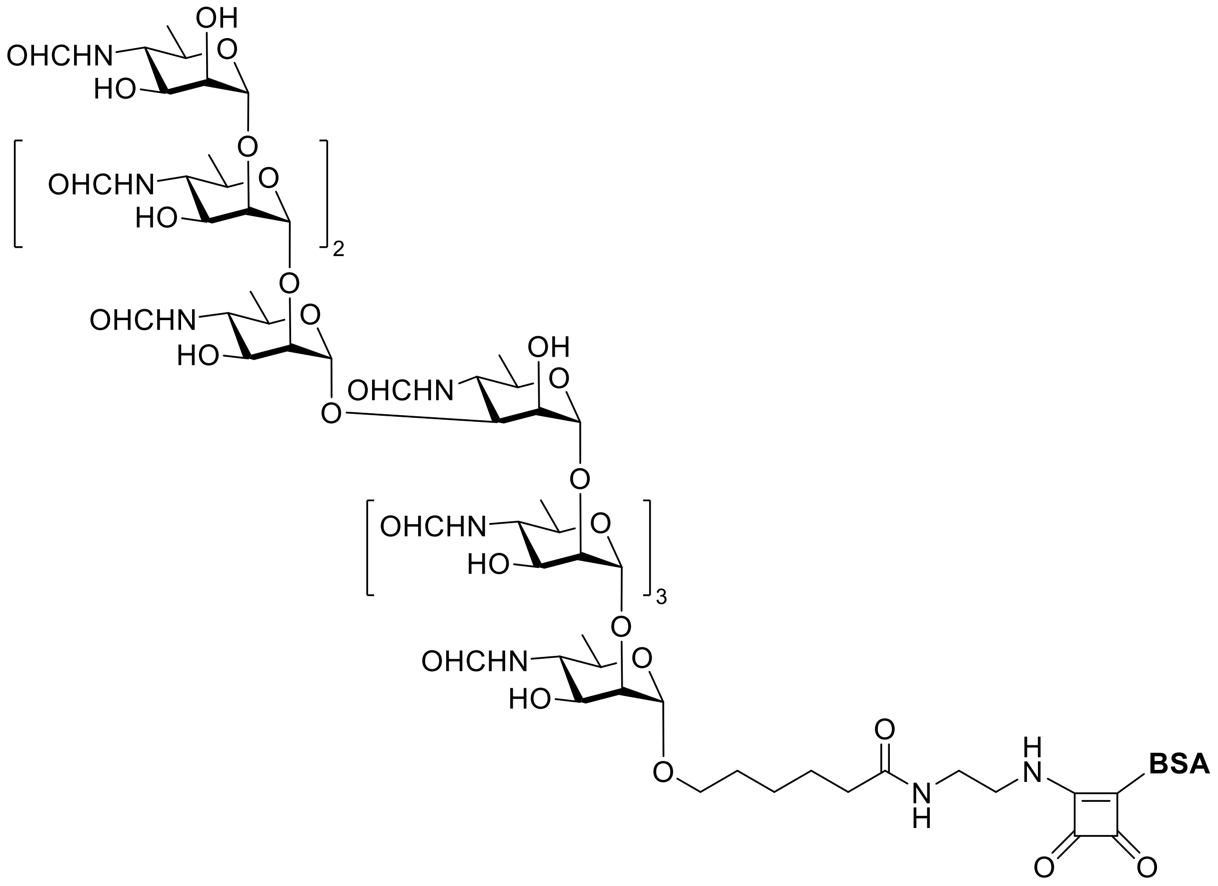

Structure **10**: 2,2,2,3,2,2,2,2-Nona (α-D-Rha4NFo-(1→2)-α-D-Rha4NFo-(1→2)-α-D-Rha4NFo-(1→2)-α-D-Rha4NFo-(1→3)- α-D-Rha4NFo-(1→2)-α-D-Rha4NFo- α-D-Rha4NFo-(1→2)-α-D-Rha4NFo-(1→2)-α-D-Rha4NFo)

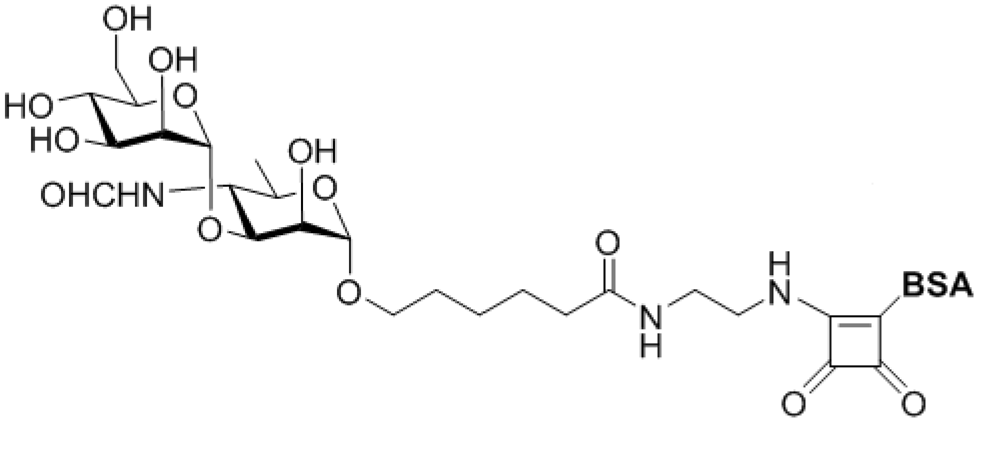

Structure **11**: Man-Mono (α-D-Man-(1→2)-α-D-Rha4NFo)

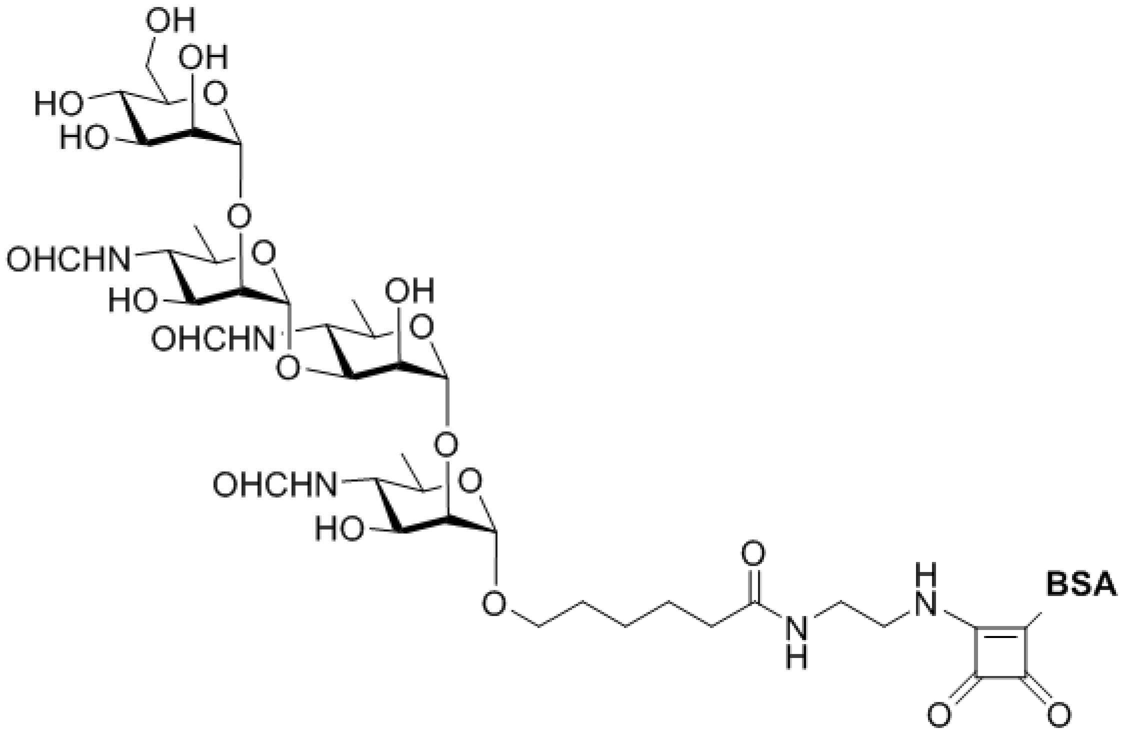

Structure **12**: Man-3,2-Tri (α-D-Man-(1→2)-α-D-Rha4NFo-(1→3)-α-D-Rha4NFo-(1→2)-α-D-Rha4NFo)

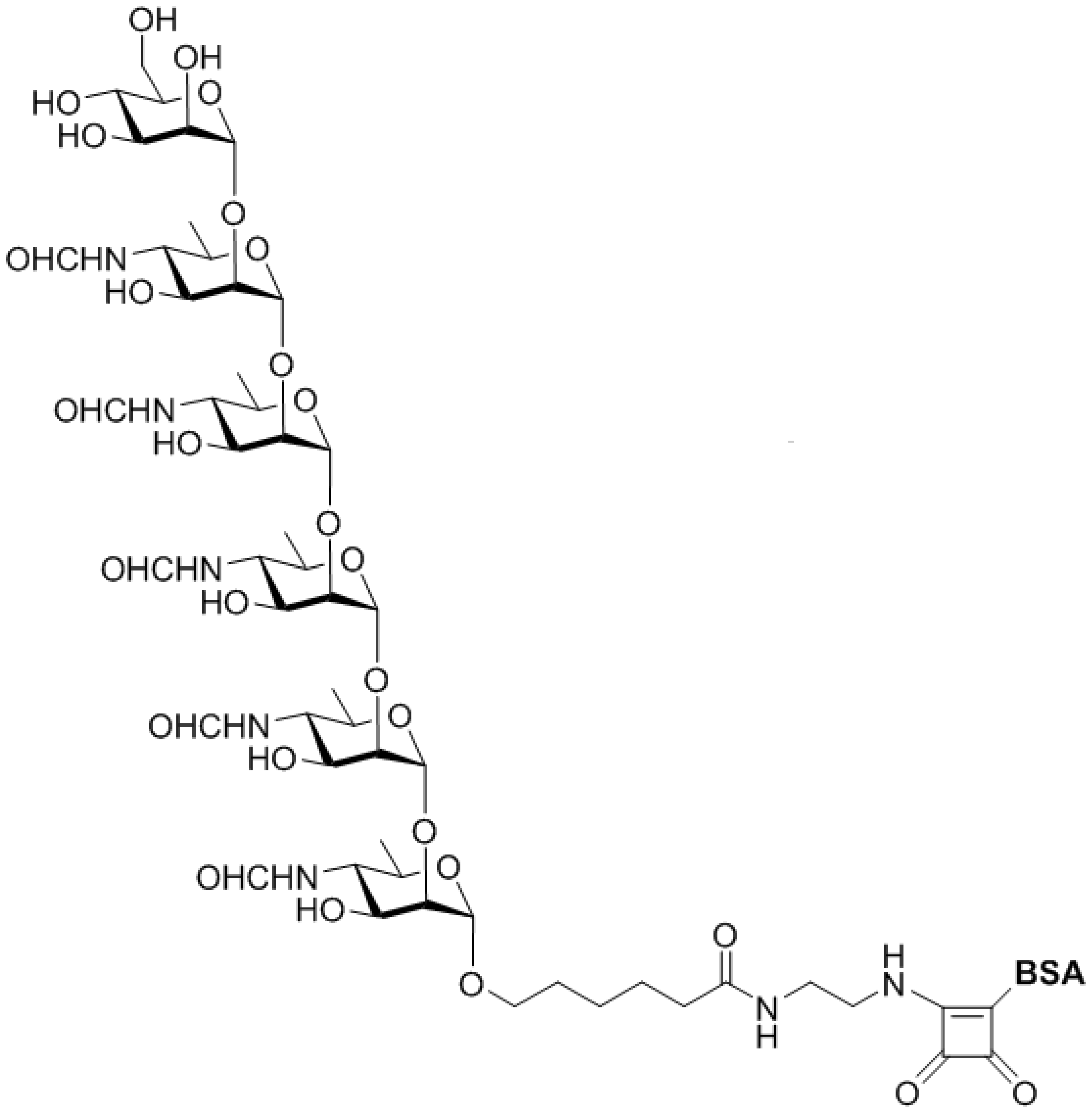

Structure **13**: Man-2,2,2,2-Penta (α-D-Man-(1→2)-α-D-Rha4NFo-(1→2)-α-D-Rha4NFo-(1→2)-α-D-Rha4NFo-(1→2)-α-D-Rha4NFo-(1→2)- α-D-Rha4NFo)

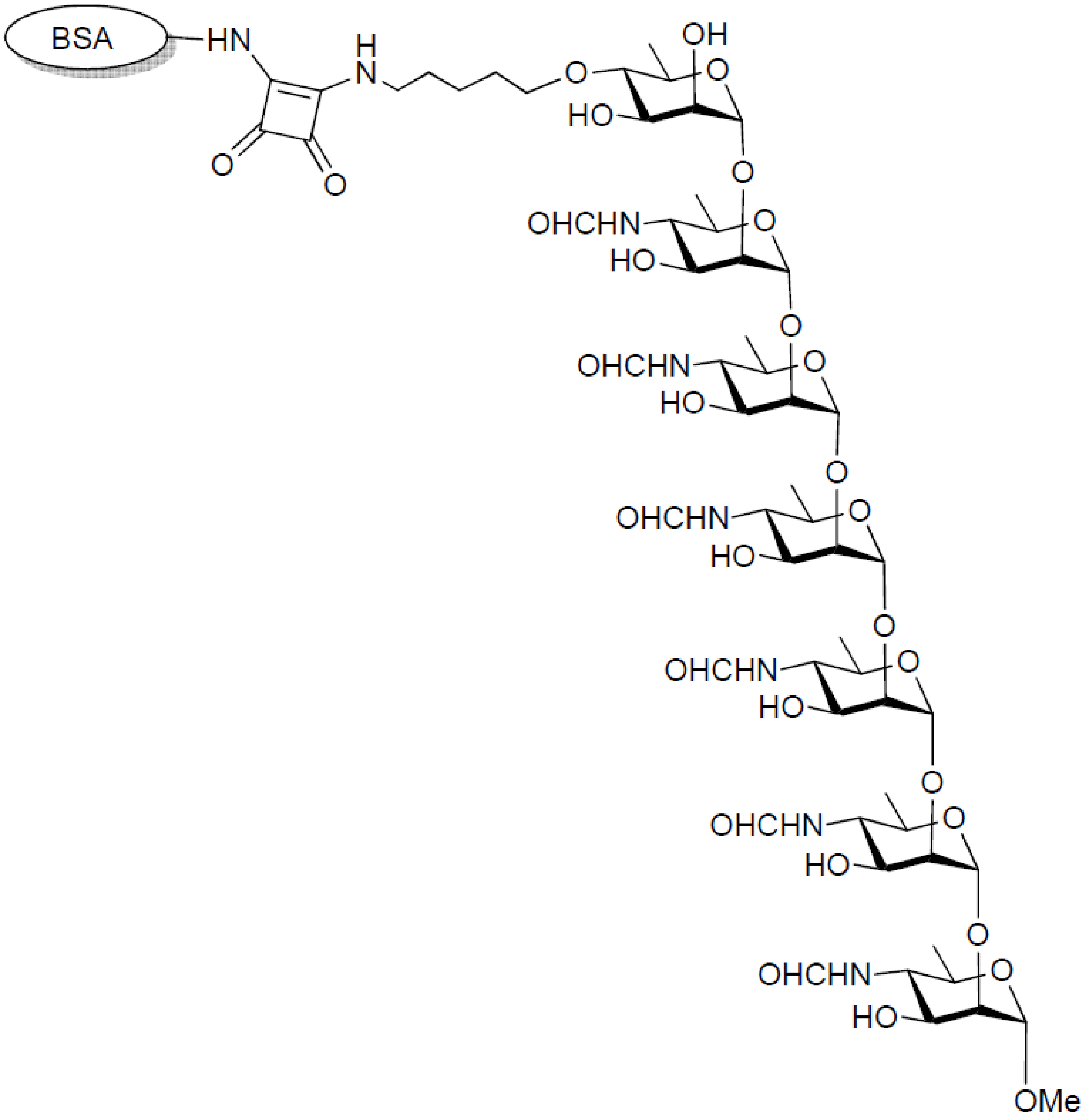

Structure **14**: Tc-2,2,2,2,2,2-Hexa (BSA-Sq-α-D-Rha-(1→2)-α-D-Rha4NFo-(1→2)-α-D-Rha4NFo-(1→2)-α-D-Rha4NFo-(1→2)-α-D-Rha4NFo-(1→2)- α-D-Rha4NFo-(1→2)-α-D-Rha4NFo)

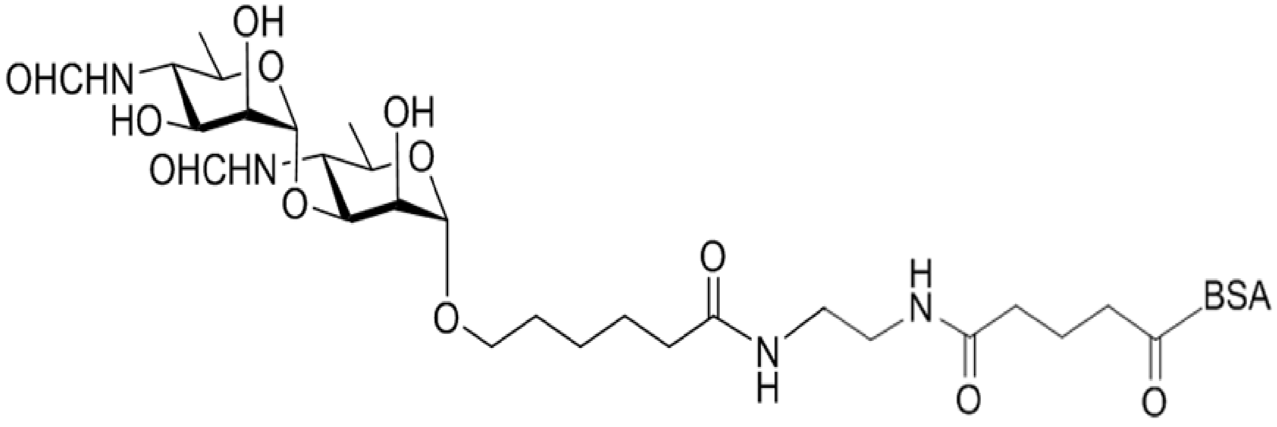

Structure **15**: 3-Di-dsg (α-D-Rha4NFo-(1→3)-α-D-Rha4NFo)

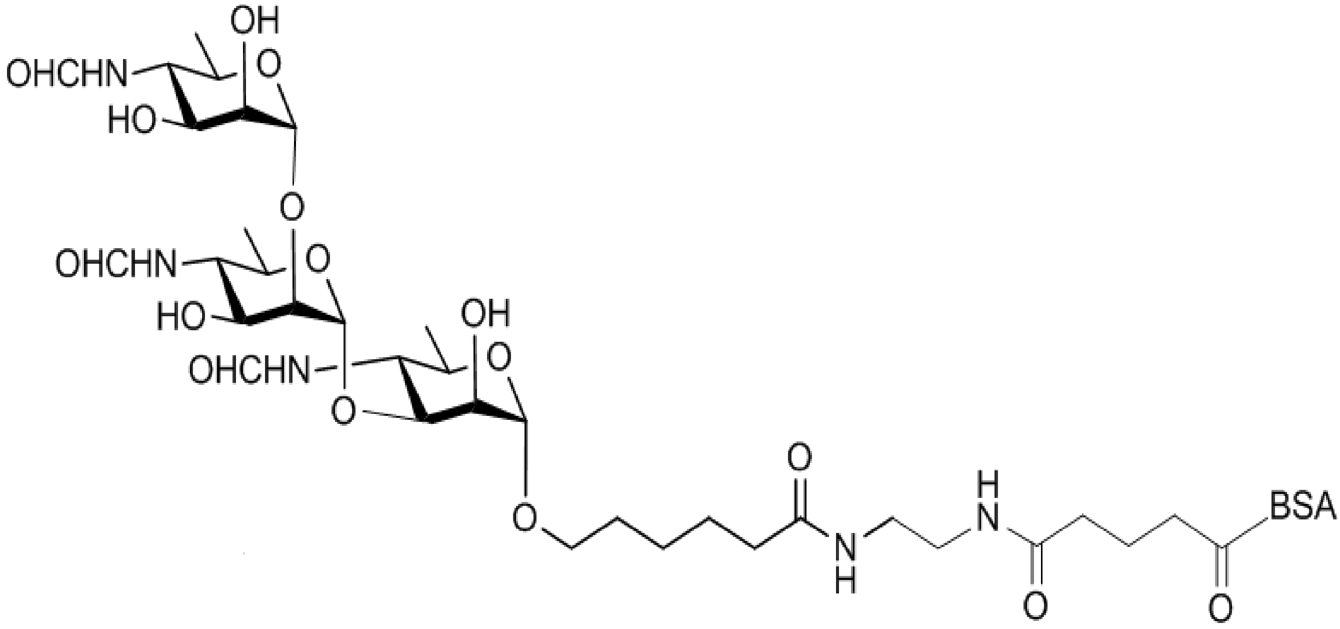

Structure **16**: 2,3-Tri-dsg (α-D-Rha4NFo-(1→2)-α-D-Rha4NFo-(1→3)-α-D-Rha4NFo)

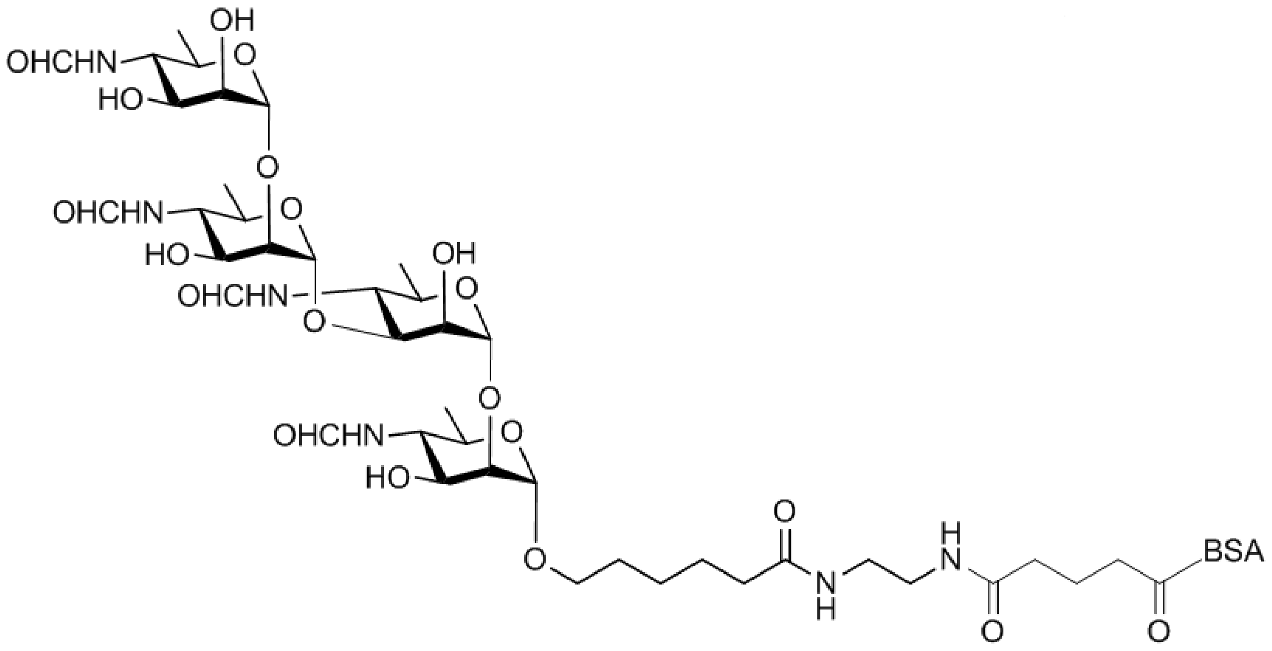

Structure **17**: 2,3,2-Tetra-dsg (α-D-Rha4NFo-(1→2)-α-D-Rha4NFo-(1→3)-α-D-Rha4NFo-(1→2)-α-D-Rha4NFo)

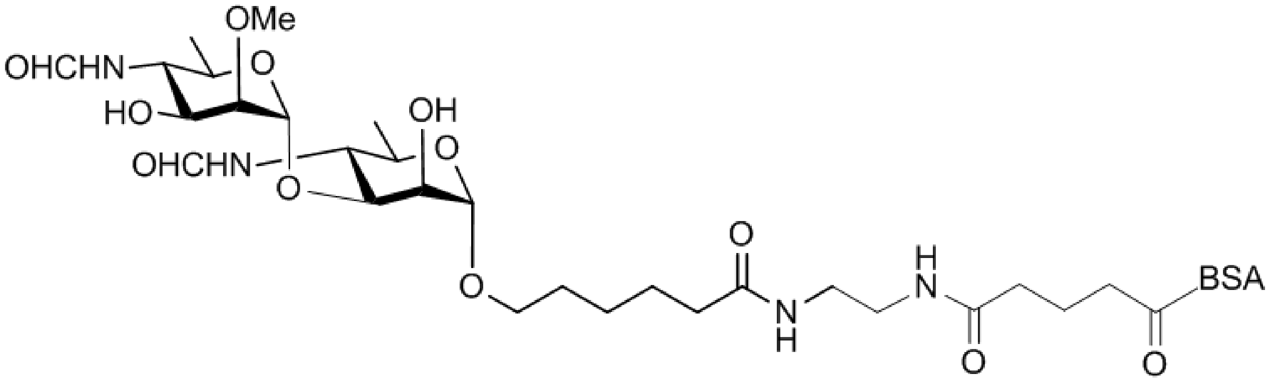

Structure **18**: 2Me-3-Di-dsg (α-D-4-6-dideoxy-4-formamido-2-O-methyl-mannopyranosyl-(1→3)-α-D-Rha4NFo)

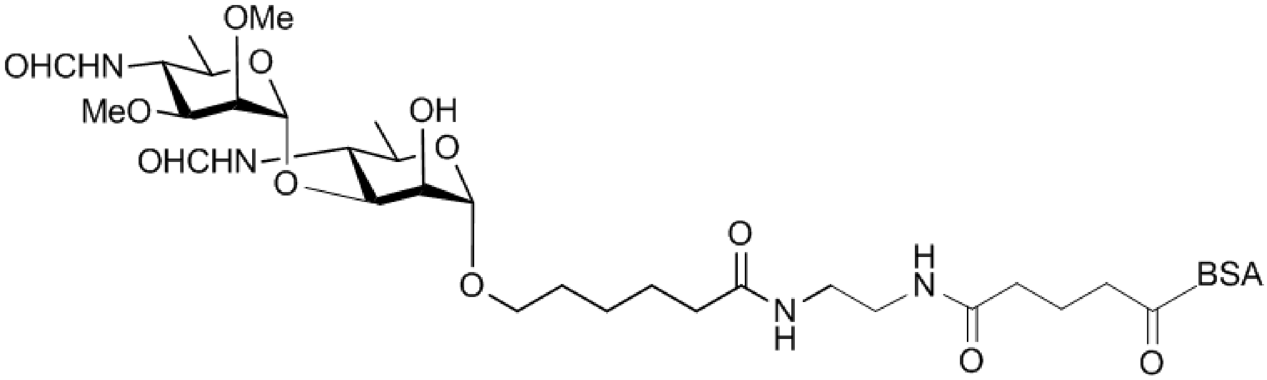

Structure **19**: 22Me-3-Di-dsg (α-D-4-6-dideoxy-4-formamido-2-3-O-methyl-mannopyranosyl-(1→3)-α-D-Rha4NFo)

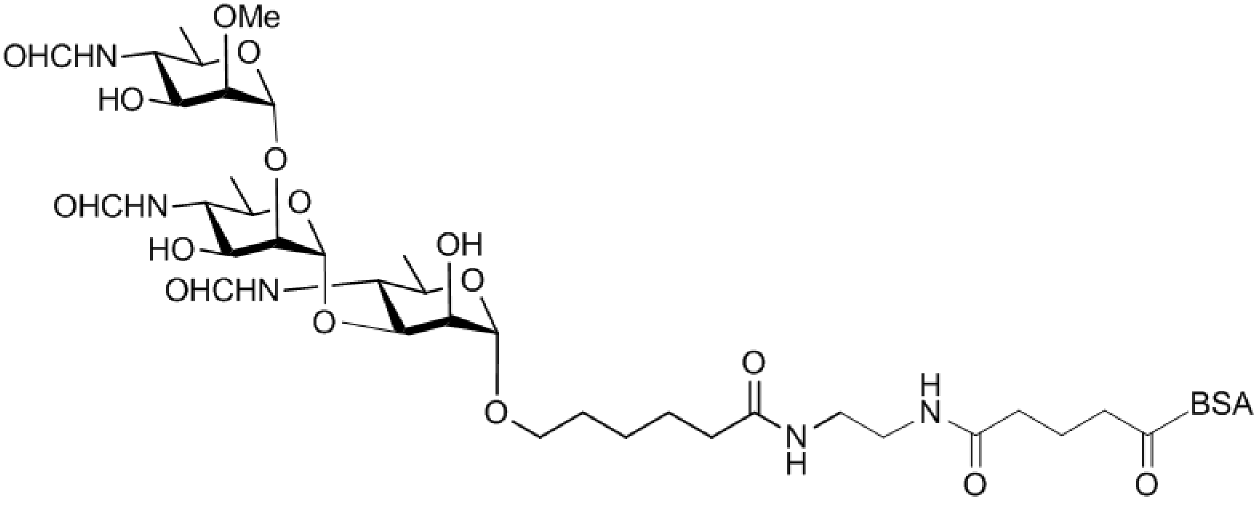

Structure **20**: 2Me-2,3-Tri-dsg (α-D-4-6-dideoxy-4-formamido-2-O-methyl-mannopyranosyl-(1→2)-α-D-Rha4NFo-(1→3)-α-D-Rha4NFo)

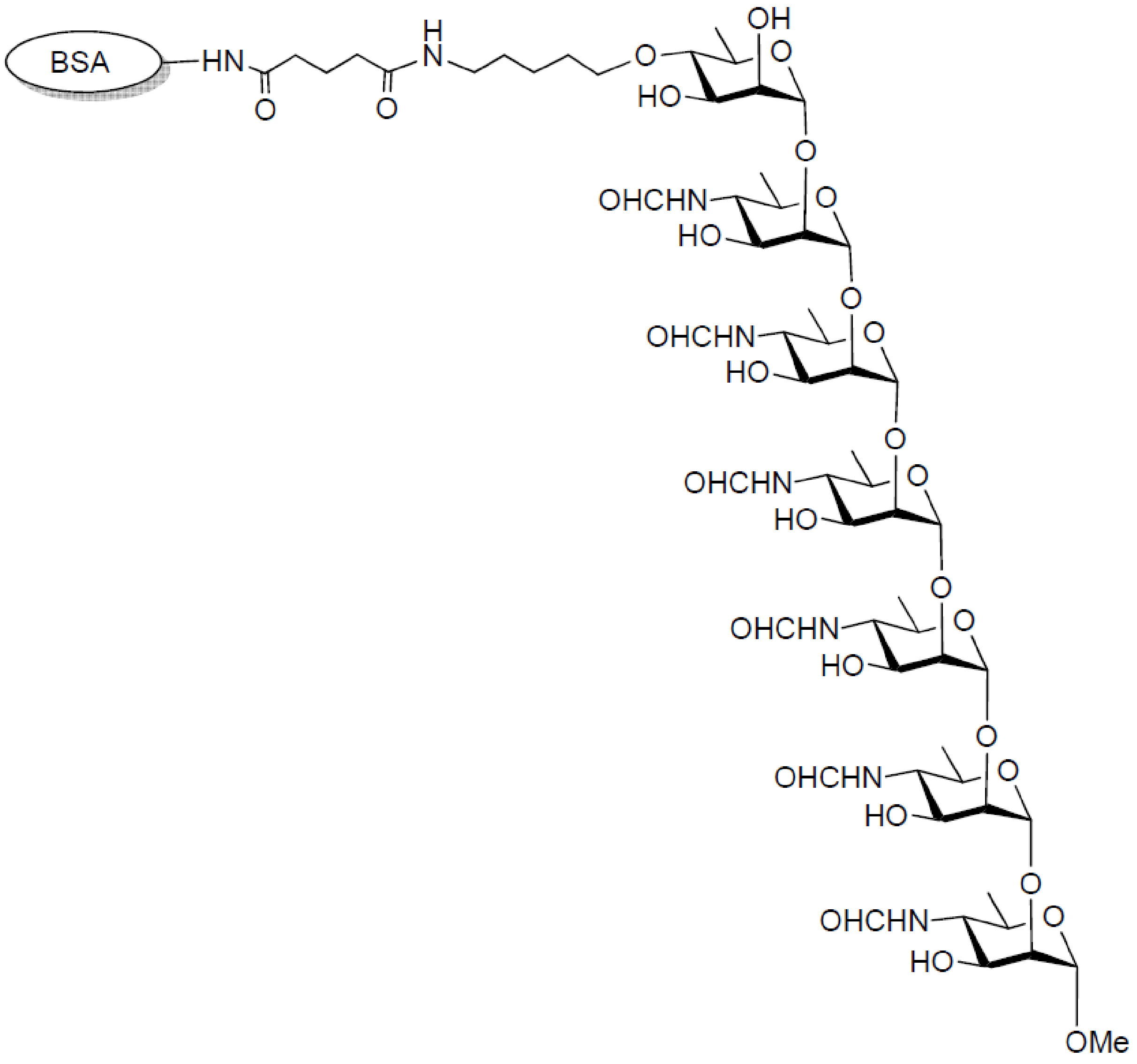

Structure **21**: Tc-2,2,2,2,2-Hexa-dsg (BSA-DSG-*α*-D-Rha-(1→2)-*α*-D-Rha4NFo-(1→2)-*α*-D-Rha4NFo-(1→2)-*α*-D-Rha4NFo-(1→2)-*α*-D-Rha4NFo-(1→2)- *α*-D-Rha4NFo-(1→2)-*α*-D-Rha4NFo)
